# Predicting 12- and 24-month documented CKD progression from routine laboratory data: a single-center temporal evaluation

**DOI:** 10.1080/0886022X.2026.2753249

**Published:** 2026-10-28

**Authors:** Mudan Jin, Jie Jing, Di Lin, Jing Li, Lang Zhang, Pinhao Guo, Zheng Wang, Xingzhu Wen

**Affiliations:** ^a^ Department of Laboratory Medicine, Shanghai Fengxian District Traditional Chinese Medicine Hospital, Shanghai, China; ^b^ Department of Laboratory and Pathology, 72nd Group Army Hospital, Huzhou, China; ^c^ Department of Laboratory Medicine, Wangjiang Subdistrict Community Healthcare Center, Shangcheng District, Hangzhou, China; ^d^ Department of Anesthesiology, 72nd Group Army Hospital, Huzhou Normal University, Huzhou, China; ^e^ Department of Hepatobiliary and Pancreatic Surgery, The Second People’s Hospital of Huai’an, the Affiliated Huai’an Hospital of Xuzhou Medical University, Huai’an, China; ^f^ Department of General Surgery, 72nd Group Army Hospital, Huzhou Normal University, Huzhou, China

**Keywords:** Chronic kidney disease, routine laboratory data, risk prediction, temporal evaluation, calibration, documentation review

## Abstract

**Background:**

Routine laboratory data may support CKD follow-up. We evaluated 12- and 24-month documented progression prediction and exploratory review outputs.

**Methods:**

This retrospective single-center study used 2012–2025 hospital records. CKD required objective persistence for ≥90 days. Development used 2012–2021 indices; temporal evaluation used unseen patients indexed in 2022–2023. Among horizon-evaluable indices, the endpoint was documented sustained ≥40% estimated glomerular filtration rate (eGFR) decline or kidney failure. Development selected 12-month and conditional second-year models combined for non-decreasing cumulative risk. Laboratory and documentation outputs were assessed separately.

**Results:**

Temporal evaluation of horizon-evaluable patients included 143 patients (10 event patients; 21/364 positive indices) at 12 months and 87 patients (22 event patients; 50/247 positive indices) at 24 months. Area under the precision–recall curve (AUPRC) was 0.510 (95% confidence interval [CI] 0.217–0.789) and 0.677 (0.459–0.853), respectively. Incremental 12-month AUPRC over age, sex and current eGFR was uncertain (difference −0.009, 95% CI −0.171 to 0.123). At 24 months, area under the receiver operating characteristic curve (AUROC) was higher than the core comparator (difference 0.057, 95% CI 0.007–0.121); clinical utility was not evaluated. Calibration slopes were 0.996 (0.689–1.640) and 1.028 (0.676–1.661); wide CIs precluded reliable individual-risk calibration. Documentation ranking did not improve on prior-mention strategies.

**Conclusions:**

This study is proof of concept. Small event counts, follow-up selection and uncertain incremental value limit interpretation. Multisite validation is required before implementation.

## Introduction

Chronic kidney disease (CKD) is common, clinically heterogeneous and associated with substantial kidney and cardiovascular morbidity worldwide [1,2]. Current guidance defines CKD by abnormalities of kidney structure or function that persist for at least three months and recommends follow-up informed by prognosis rather than stage alone [3,4]. Established kidney-failure equations support risk stratification from routinely measured variables [5–7], while dynamic models show that updated laboratory measurements can revise risk beyond a single baseline assessment [8].

Risk estimation is only one part of a CKD follow-up encounter. Clinicians also review current laboratory abnormalities and existing documentation. These tasks have different targets: a progression model estimates a future recorded outcome; a laboratory rule identifies a measured abnormality; and a documentation model predicts which disease terms are recorded, not whether the patient truly has that disease. Routine laboratory information systems (LIS) can support these tasks, provided their evidence boundaries and missingness are explicit [9,10].

Our primary question was whether routine information available at a CKD follow-up encounter could predict documented progression within 12 and 24 months in previously unseen patients from a later period at the same hospital. We assessed incremental performance beyond age, sex and current eGFR and imposed non-decreasing risk across horizons by combining 12-month and conditional second-year probabilities. A secondary, exploratory objective was to describe current laboratory states and prioritize locally recorded documentation targets, independently of progression prediction. These analyses assess a research-stage framework and do not define a clinical action threshold or support deployment.

## Materials and methods

### Study design, setting and source data

This retrospective single-center prediction-model study was organized around an eligible CKD follow-up index. From the same history frozen at that index, the analysis generated two prognostic components and a separate current-review output. The source comprised 1,549,924 raw LIS rows from Shanghai Fengxian District Traditional Chinese Medicine Hospital, Shanghai, from 13 December 2012 through 31 December 2025. Prespecified kidney-related test and diagnosis filters retained 1,549,903 rows for linkage and cohort construction. Each row contained collection and report times, typed patient identifiers, demographics, diagnosis text, test name, result and unit. Report time defined when a value became available to prediction. Reporting follows the Transparent Reporting of a multivariable prediction model for Individual Prognosis Or Diagnosis + Artificial Intelligence (TRIPOD+AI) and REporting of studies Conducted using Observational Routinely-collected health Data (RECORD) principles [9,10]. Assay-level quality-control retention and current-state predictor availability are summarized in Supplementary Figure S1.

### Identity linkage

Records were linked by transitive exact matching within identifier type. A shared national identification number, social-insurance/outpatient identifier, or inpatient/medical-record identifier linked two records to the same person; values from different identifier types were never equated merely because their strings matched. Connected records formed a patient component. Components with discordant recorded sex were excluded from the primary cohort when the conflict persisted after linkage quality checks. Names were not used as linkage keys.

### Inclusion and exclusion criteria

At each encounter, an internally reported eGFR between 1 and 200 mL/min/1.73 m^2^ was used when available. Otherwise, eGFR was calculated from serum creatinine, age and sex with the 2021 Chronic Kidney Disease Epidemiology Collaboration (CKD-EPI) creatinine equation without race [11]. Calculated eGFR was withheld when age or a consistent recorded sex was unavailable. This same eGFR hierarchy was used for CKD confirmation, prediction-index eligibility, outcome ascertainment and eGFR-derived predictors.

At retained indices, reported eGFR was used for 14/1,463 development indices (1.0%) and 368/563 primary temporal indices (65.4%); all remaining values were calculated. These are index-level proportions, not proportions of all source assays. Historical laboratory equation identifiers were not available in the analytical extract. Paired reported-versus-calculated values, GFR-category agreement and the complete uniform-equation sensitivity are reported below.

All inclusion requirements were applied before prediction-index construction: a linkable typed identifier; age 18–100 years at the candidate encounter; at least one explicit serum-creatinine assay with a numeric result of 20–2,000 μmol/L, an interpretable concentration unit, valid collection and report times, and reporting from collection through seven days afterwards; objective CKD evidence on at least two dates separated by at least 90 days within the same evidence domain; and a subsequent creatinine-based encounter after CKD confirmation. CKD was confirmed on the earliest second evidence date when both dates showed eGFR below 60 mL/min/1.73 m^2^ or both showed qualifying proteinuria. Qualifying proteinuria was urine dipstick at least 1+ (trace and weak-positive results excluded), 24-h protein at least 150 mg/day, or at least 0.15 g/day with an interpretable 24-h unit. Diagnosis text alone could not establish CKD.

Failure to satisfy any inclusion requirement was treated as screening failure and was not counted again as a post-inclusion exclusion. Among patients with objectively confirmed CKD, we excluded unresolved sex-discordant linkage components, patients without a subsequent valid age- and eGFR-resolved creatinine encounter, and patients with no eligible outcome-free encounter in either prespecified analysis period. Candidate encounters on or after confirmed kidney-failure onset were excluded. Pre-index kidney failure comprised recorded uremia, end-stage kidney disease/end-stage renal disease (ESKD/ESRD), maintenance dialysis, kidney transplantation or sustained eGFR below 15 mL/min/1.73 m^2^ on two dates at least 30 days apart. Separately, a single candidate eGFR below 15 was a prespecified CKD G5 safety gate that suppressed prediction at that encounter; it was not classified as confirmed kidney failure. These deterministic gates were applied before outcome labels, features or models were constructed. The complete eligibility and prediction-index sequence is shown in Figure 1.

**Figure 1.**
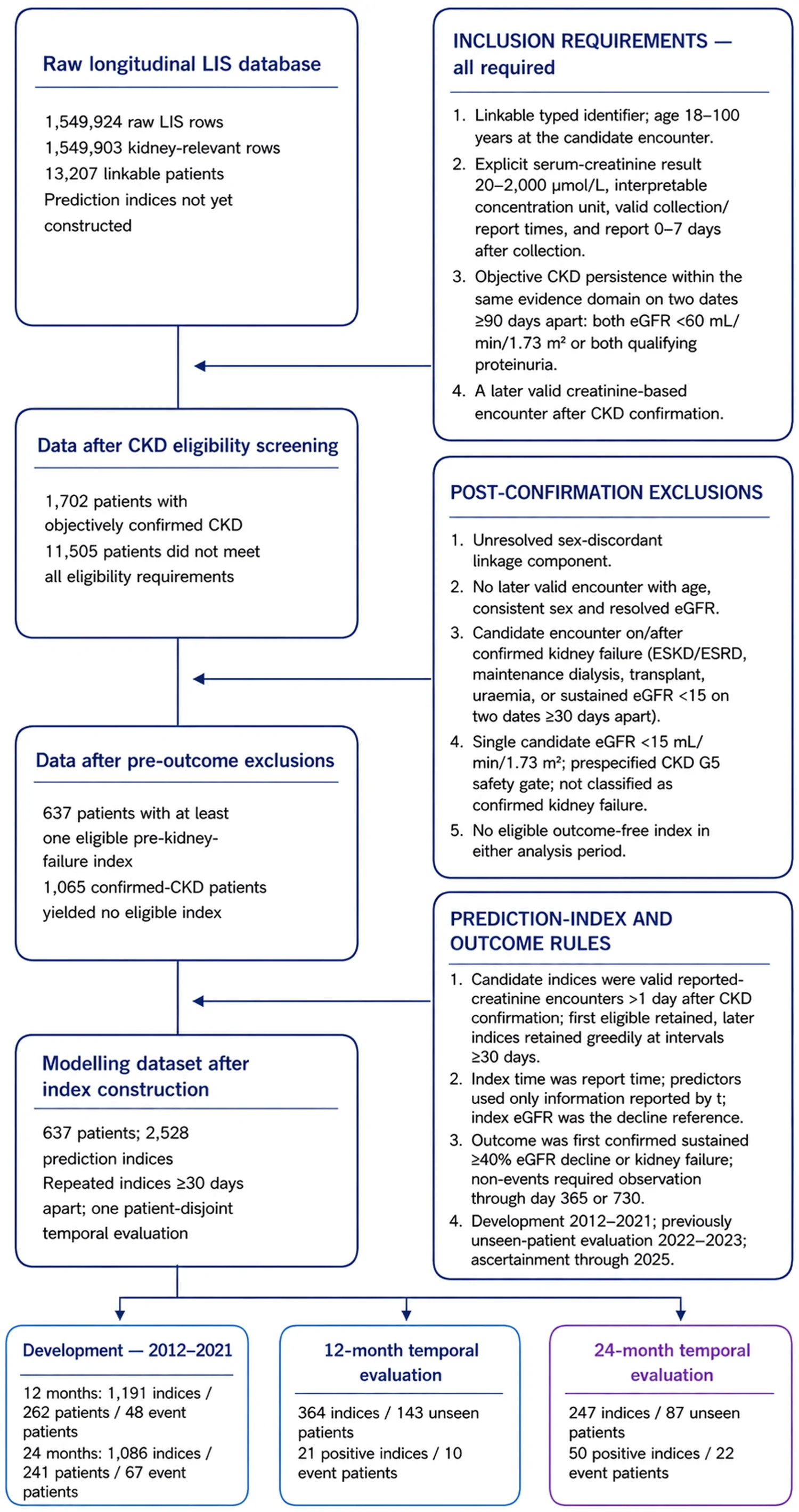
Cohort construction, eligibility gates, prediction-index construction and horizon-specific samples. Screening failures are distinct from exclusions applied after objective CKD confirmation. Each criteria arrow terminates on the connector between the data states to which its rule set applies. The primary temporal cohort excluded 144 later-period patients already represented in development.

### Prediction-index construction and temporal split

CKD confirmation itself was not used as a prediction index. For each patient, candidate indices were valid reported-serum-creatinine encounters occurring more than one day after confirmation, with age, sex-consistent eGFR and report time available and no kidney failure on or before that encounter. Within each analysis period, encounters were ordered chronologically; the first eligible encounter was retained and subsequent encounters were retained only when at least 30 days had elapsed since the previously selected index. This deterministic greedy spacing was independent of subsequent outcomes. The index time was the creatinine report time, and only records reported at or before that time contributed predictors. Each index eGFR served as the reference value for subsequent percentage decline.

A patient is a linked individual; an index is one eligible creatinine report-time prediction occasion. A positive index has documented progression within its own horizon. An event patient has at least one positive index at that horizon and may also contribute nonevent indices. Consequently, positive-index counts do not represent independent events. Time zero is each index report time, the historical summary window is the preceding 365 days, and the prediction windows end at days 365 and 730. Current-state values are the latest available by time zero; laboratory review rules require index-date collection and availability by time zero.

Repeated indices represented the intended research use of updating predictions when new creatinine results become available during follow-up. The 30-day spacing was a pragmatic rule to reduce near-duplicate assessments; it was not a validated clinical review interval, and overlapping histories remain correlated. All indices of one patient stayed in the same development fold or bootstrap cluster. Inverse index-frequency weights gave each patient equal total weight within the relevant analysis sample, while averaging over that patient’s eligible encounters. The estimand therefore differs from prediction at one baseline encounter. The single-landmark sensitivity retains the earliest horizon-evaluable index per patient. Index distributions and proximity to event confirmation are reported with the observation-frequency analyses below.

Development indices were dated from 13 December 2012 through 31 December 2021. This outcome-independent cutoff was fixed before modeling and left the remaining database period for maturation of 24-month outcomes. Primary temporal evaluation used indices from 1 January 2022 through 31 December 2023, with outcome ascertainment through 31 December 2025. Every patient represented in development was removed from primary temporal evaluation before horizon-specific evaluability was assessed. A prespecified stricter-gap sensitivity analysis ended development on 31 December 2020 and evaluated indices dated in 2023.

### Outcome definition and horizon evaluability

The primary outcome was first documented confirmed CKD progression within the available hospital record system after the index: sustained at least 40% eGFR decline from the index eGFR or kidney failure, whichever occurred first. Sustained decline required two qualifying post-index measurements at least 30 days apart; its event date was the second qualifying measurement. A 40% decline was selected because confirmed eGFR decline has established prognostic relevance and is commonly used in CKD progression studies [12,13]. Kidney failure comprised recorded uremia/ESKD/ESRD, maintenance dialysis, kidney transplantation, or sustained eGFR below 15 on two post-index dates at least 30 days apart. The mutually exclusive outcome component was assigned by the earliest qualifying date; kidney failure took precedence if both components shared that date. Kidney failure alone was prespecified as a sensitivity endpoint.

An index was positive at a horizon if the first outcome occurred by day 365 or day 730. A nonevent required a valid kidney-function measurement at or after the horizon end; a later unrelated LIS record was insufficient. Thus, all 24-month-evaluable indices were also evaluable at 12 months, but not all 12-month indices had sufficient follow-up for 24 months.

An index without a documented event by the horizon and without a sufficiently late kidney-function measurement was non-evaluable, not a nonevent. The evaluated estimand is progression recorded by the horizon conditional on available predictors and horizon evaluability. It is not the unconditional risk among all patients presenting for follow-up. This binary design gives explicit 12- and 24-month labels for the available records, at the cost of discarding incompletely observed indices. A censoring-based landmark survival or discrete-time model could retain such indices, but would require an explicit observation/censoring model and assumptions about unobserved events. Death dates were unavailable: death could not be coded as a competing event. If an unrecorded death prevented sufficient subsequent testing and no progression was recorded, the index remained non-evaluable.

### Separate 12- and 24-month risk tasks and predictor families

We modeled 12-month cumulative risk p12 = P(*T* ≤ 365|Xt,E12 = 1) and conditional second-year risk q13–24 = P(365<*T* ≤ 730|*T* > 365,Xt,E24 = 1). The p12 model used 12-month-evaluable development indices. The conditional model was trained only on 24-month-evaluable development indices that remained outcome-free through day 365; its target was first progression during days 366–730. During cross-validation, folds were assigned on the complete 24-month sample before early-event rows were removed from conditional-model training, ensuring that no validation patient contributed to fitting. The conditional model generated q13–24 for every 24-month-evaluable index so that the coherent cumulative output could be calculated as p24 = p12 + (1 − p12)q13–24. This identity guarantees p24 ≥ p12 for every index. A directly fitted 24-month model was prespecified as a sensitivity comparator, not as the primary output.

More precisely, the fitted binary components estimate risks conditional on their respective evaluability sets: p12 approximates P(Y12 = 1 | X, E12 = 1), and q13–24 approximates P(Y24 = 1 | Y12 = 0, X, E24 = 1), where Eh denotes evaluability at horizon h. Because these selected risk sets differ, algebraic composition guarantees ordering but does not itself establish calibration or identify unconditional cumulative risk in the full presenting population. Survival and discrete-time frameworks instead construct cumulative risks from a common time-to-event process; their assumptions would need separate assessment in these intermittently observed records.

Three prespecified predictor families entered the development-only grid. The core family contained age, sex and current eGFR. The current-state family added the latest hemoglobin, albumin, potassium, phosphate and urea values reported by the index. The expanded longitudinal family further added the median, ordinary least-squares slope, standard deviation and measurement count for each analyte during the preceding 365 days. Both risk components had to use the same feature family, although their algorithms could differ. The current-state family was the prespecified practical candidate; the expanded family could replace it only if its optimized pipeline increased development AUPRC without worsening Brier score. The core family remained a common clinical benchmark and could not be substituted after temporal evaluation. Diagnosis text, cue states and review-cue labels were excluded from the primary risk models. Figure 2 summarizes the separation of the risk and same-day review pathways.

**Figure 2.**
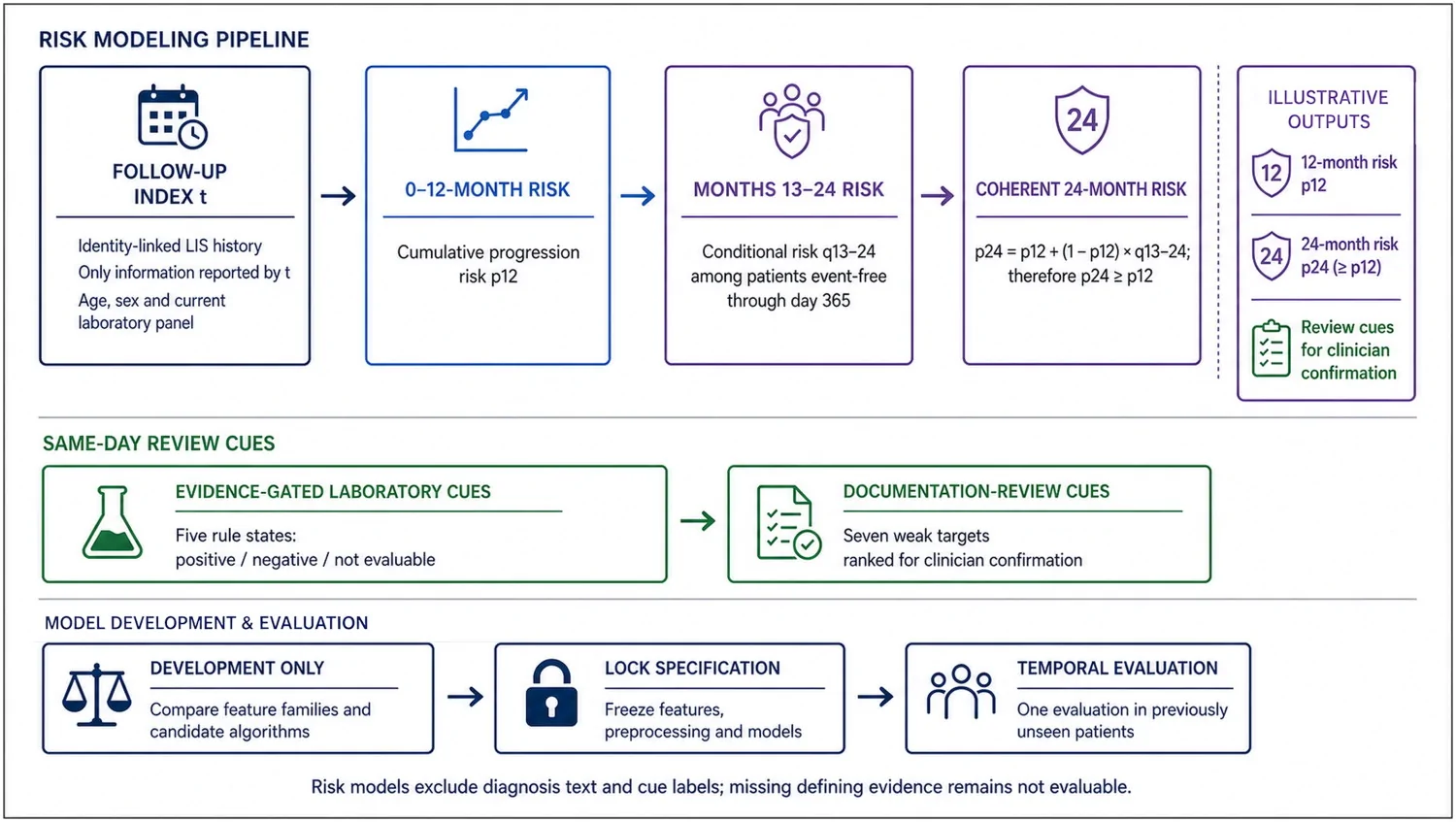
Study architecture. The risk pathway uses the current-state laboratory family locked by the single development-only joint candidate grid. A 0–12-month cumulative component and conditional months 13–24 component yield coherent cumulative 12- and 24-month risks. Separate laboratory and documentation-review cue pathways do not enter either risk model; missing defining evidence remains not evaluable rather than negative.

### Same-day review-cue construction

The review-cue module was not used to predict future progression. Five laboratory-defined cues were generated by prespecified rules and displayed as positive, negative or not evaluable: CKD G4 (index eGFR 15–29.9 mL/min/1.73 m^2^), anemia in CKD (hemoglobin collected on the index date and reported by the prediction time <130 g/L in men or <120 g/L in women), hyperphosphatemia (phosphate >1.45 mmol/L), hyperkalemia (potassium >5.5 mmol/L), and proteinuria using the qualifying urine rules above. Only results collected on the index date and reported by the prediction time were eligible. CKD G5 was a pre-model safety gate; risk scores were suppressed when index eGFR was below 15. Missing defining evidence produced not evaluable, never a negative cue. Three-state proportions were descriptive and were not interpreted as diagnostic performance.

Seven kidney-disease documentation targets were weakly labeled from prespecified patterns in diagnosis text collected on the index date and reported by the prediction time: diabetic kidney disease, hypertensive nephropathy, glomerular disease, IgA nephropathy, nephrotic syndrome, polycystic kidney disease and obstructive nephropathy. A target was evaluable only when non-empty index-date text was available; no pattern indicated no documented mention, not confirmed absence of disease. Predictors included age, sex, current laboratory values and, for each target, any prior mention and the number of distinct mention dates during the preceding 365 days. Only pre-index diagnosis records were used as predictors; index-date text was reserved for target construction. The model ranked targets for clinician review rather than estimating the probability of true CKD etiology. Supplementary Table S1 summarizes the laboratory rules and labels; the restricted-access Zenodo reproducibility package (DOI: 10.5281/zenodo.23039194) provides the exact patterns and evaluation boundaries (documentation/Documentation_Label_Rules.md and src/multilabel.py).

### Development-only joint pipeline selection and locking

Risk-pipeline selection used a joint grid confined to development data. Within the core, current-state and expanded longitudinal families, four prespecified algorithms (L2-penalized logistic regression, elastic-net logistic regression, random forest and histogram-based gradient boosting) were fitted separately for 12-month cumulative risk and conditional months 13–24 risk, yielding 24 candidate task configurations. Five repeats of five-fold stratified patient-group cross-validation were used. AUPRC was primary because outcomes were imbalanced [14]. Within each family and component, the algorithm with the highest AUPRC was selected; differences within 0.02 were resolved by lower Brier score and then the prespecified simplicity rule. The two selected components within each family were composed, and the shared-family pipelines were compared using cumulative-horizon AUPRC and Brier criteria. The expanded family could replace the current-state family only if AUPRC improved without a worse Brier score. Imputation, scaling and hyperparameter choices were fitted within each training fold. This procedure locked the current-state elastic-net 12-month model and the current-state random-forest conditional model. Their composition defined the primary 24-month output; no further model choice was made. The direct 24-month sensitivity model followed the same development-only rule.

Because the same development sample was used to select and evaluate the pipeline, the repeated grouped out-of-fold estimates are not selection-adjusted internal validation. A supplementary audit reapplied the prespecified rules within each repeat to examine selection consistency. This audit does not replace nested validation, bootstrap assessment of the full selection procedure or external replication; repeat-level results are reported below.

For the seven weakly supervised documentation targets, each algorithm family fitted one binary classifier per development-estimable label. Model comparison used three repeats of patient-grouped cross-validation, with up to five folds and fewer folds when the number of positive or negative patients required it. The primary selection metric was macro-AUPRC across development-estimable labels; candidates within 0.01 of the maximum were resolved by higher mAP@3 and then prespecified simplicity. A label was development-estimable only when at least five positive and five negative patients were available.

After the shared feature family and task-specific algorithms had been selected using development data alone, preprocessing and each locked model were refitted on all task-eligible development indices. The conditional model was refitted on all 24-month-evaluable development indices that were outcome-free through day 365, and each documentation-label classifier was refitted on all evaluable development indices for that label. Frozen models were then applied once to the patient-disjoint temporal cohort without model reselection, hyperparameter tuning or recalibration. Frozen hyperparameters are reported in Supplementary Table S2. Complete metrics for all 24 risk-task configurations and the resulting family-level coherent pipelines are reported in Supplementary Table S3 and Supplementary Figure S2. Supplementary Table S4 provides the methods–results crosswalk.

### Descriptive statistics and predictor availability

Baseline characteristics were summarized at each patient’s earliest eligible index in the development or primary temporal split. Continuous variables are reported as median and interquartile range, categorical variables as n (%), and missingness as the percentage of patients without a latest value at that index. No between-cohort hypothesis tests were performed. Assay-level quality-control retention and predictor availability are detailed in Supplementary Figure S1.

### Risk-model performance, incremental comparison and uncertainty

Risk-model performance was summarized by patient-weighted AUPRC (primary), AUROC and Brier score. Development comparisons used the joint feature-family and algorithm grid described above. For the temporal increment analysis, the fixed clinical reference used age, sex and current eGFR in an L2-penalized logistic model. Median imputation, available missingness indicators, standardization and patient-weighted fitting used task-eligible development indices, and the fitted transformations were applied unchanged to the same temporal evaluation indices. The reference was neither selected nor modified using temporal results (the restricted-access Zenodo reproducibility package (DOI: 10.5281/zenodo.23039194), documentation/Clinical_Reference_Implementation.md). It quantified incremental information beyond a simple clinical benchmark and could not replace the locked model *post hoc*. Paired differences in AUPRC, AUROC and Brier score used the same 2,000 patient-cluster bootstrap samples; Brier improvement was reference minus locked model. The clinical core was an empirical benchmark, not a substitute for the Kidney Failure Risk Equation (KFRE) or guideline-recommended assessment of GFR and albuminuria [3,5–7]. Calibration followed published guidance [15]. We did not undertake decision-curve analysis or threshold claims because no clinical action threshold or intervention consequence had been specified [16]. All eligible records in the fixed calendar periods were included. No separate *a priori* sample-size calculation was performed; event-patient counts and cluster confidence intervals are therefore reported to characterize precision.

Additional analyses are indexed in numerical order: horizon evaluability (Supplementary Table S5), index frequency and event proximity (Supplementary Table S6), case mix (Supplementary Table S7), predictor missingness and associations (Supplementary Tables S8 and S9), eGFR source and agreement (Supplementary Table S10), calibration (Supplementary Table S11), bootstrap event information (Supplementary Table S12), complete-case performance (Supplementary Table S13), documentation comparators (Supplementary Table S14), imputation sensitivity (Supplementary Table S15), the clinical-history comparator (Supplementary Table S16), urine albumin-to-creatinine ratio (UACR) availability (Supplementary Table S17), the Tangri comparison (Supplementary Table S18), selection stability (Supplementary Table S19) and external-validation planning (Supplementary Table S20). We estimated calibration-in-the-large (an intercept with logit(prediction) as an offset), a jointly fitted calibration intercept and slope, and a patient-weighted quartile absolute calibration error. The last measure is the weighted average absolute observed-minus-predicted difference over four prediction bins; it is not a smoothed integrated calibration index. Estimates and percentile confidence intervals used 2,000 patient-cluster bootstrap samples, retaining every selected patient’s indices and giving each resampled copy equal total weight. Model comparisons used paired cluster draws. The conditional model was evaluated only among 24-month-evaluable indices that were event-free through day 365. These fits assessed calibration and were not applied to recalibrate the frozen predictions (Supplementary Table S11).

Additional analyses described evaluability, index frequency, GFR categories, predictor missingness and associations of missingness with age, eGFR and outcome. A fixed clinical-history proxy comparator added prior diabetes, hypertension and kidney-disease mentions and index-date proteinuria positive/not-evaluable indicators to the core predictors; separate L2-logistic models (*C* = 0.2) were fitted by horizon in development data. Absent diagnoses were not treated as absent comorbidity, and validated structured data on medication, cardiovascular disease, referral and follow-up specialty were unavailable. Missing-data analyses retained the locked algorithms and hyperparameters while removing missingness indicators or restricting fitting to complete cases, without temporal reselection. We also applied the published coefficients of Tangri et al.’s three-variable sequential model 2 as a fixed linear predictor: log(0.75)×age/10 + log(1.46)×male sex + log(0.57)×eGFR/5 [5]. Because a deployable horizon-specific baseline-risk equation was unavailable and its treated-kidney-failure endpoint differs from ours, comparison was limited to AUPRC and AUROC, including the original eGFR range of 10–59 mL/min/1.73 m^2^. A separate audit reapplied the frozen selection rule within each cross-validation repeat. None of these analyses altered the locked models (Supplementary Tables S5–S20).

### Same-day review-cue performance

For documentation ranking, macro-AUPRC was the unweighted mean of label-wise average precision across temporally estimable labels; micro-AUPRC pooled their evaluable patient-label pairs. Patient-weighted mean average precision at k (mAP@k) and recall@k were calculated among indices with at least one positive estimable documentation target. Patient-label pairs without non-empty index-date diagnosis text were excluded. Label-wise temporal results required at least five positive and five negative temporal patients. Only three labels satisfied this rule: Top-3 therefore included every evaluated label, making recall@3 = 1 mechanically guaranteed rather than evidence of ranking accuracy. Top-1 and Top-2 are reported with this restriction explicit. Additional comparators ranked the same labels by development prevalence, any prior mention, or distinct mention dates in the preceding 365 days. Ties in history comparators used development prevalence; same-day text was excluded from predictors. Paired confidence intervals used 2,000 patient-cluster samples (Supplementary Table S14).

### Model explanation, synthetic illustration and software

Tree-based SHapley Additive exPlanations (TreeSHAP) summarized the selected tree model and standardized coefficients summarized the selected elastic-net model; explanations were treated as fitted associations, not causal effects [17]. For the interface illustration, we constructed a synthetic non-patient case using rounded, clinically coherent laboratory values within the observed ranges of the temporal cohort. Illustrative 12- and 24-month scores were constrained to the unit interval and to a 24-month value not lower than the 12-month value; laboratory cue states followed the prespecified rules. The example was not used for model fitting, validation, performance estimation or subgroup analysis and contains no patient-level record. The display is a conceptual prototype and has not undergone usability testing, external validation, threshold assessment, clinical-impact analysis or prospective workflow evaluation.

Analyses used Python 3.12.13 with pandas 3.0.5, NumPy 2.5.2, SciPy 1.18.0, scikit-learn 1.9.0, SHAP 0.48.0 and Matplotlib 3.11.1. Package versions and a locked environment specification are included in the reproducibility package. Additional analyses used Python 3.12.14 and reused the frozen cohort and predictions; fixed-specification refits reproduced the original probabilities to numerical precision before sensitivity analyses. Their code and aggregate outputs are included in the restricted-access Zenodo reproducibility package (DOI: 10.5281/zenodo.23039194).

### Sensitivity and information-boundary analyses

Prespecified sensitivity analyses used the stricter temporal gap, one index per patient, a kidney-failure-only endpoint, a direct 24-month model and a uniform eGFR definition. The single-landmark analysis retained each patient’s earliest horizon-evaluable index. Kidney-failure-only performance reused the corresponding locked primary predictions without model reselection. The direct 24-month model was selected by the same development-only rule as the primary risk components, and horizon reversals were counted when its cross-fitted 24-month estimate was lower than the corresponding cross-fitted 12-month estimate. To test calendar-related measurement drift, cohort construction, outcome ascertainment, predictor generation, and model selection and fitting were repeated from the source records using the 2021 CKD-EPI creatinine equation for every eligible eGFR value, irrespective of whether an internally reported eGFR was available. For each horizon, we retained each patient’s earliest evaluable temporal index within each cue-positive stratum. Estimability required at least five event and five nonevent patients at that horizon; the subgroup display reports these flags, not discrimination estimates.

### Ethics

The study followed the Declaration of Helsinki and general principles of ethical clinical research [18] and was approved by the Medical Ethics Committee of Shanghai Fengxian District Traditional Chinese Medicine Hospital (approval number: 2023042607). Because only de-identified historical clinical data were used and no clinical workflow was altered, the requirement for written informed consent was waived.

## Results

### Cohort construction and horizon-specific samples

The deterministic eligibility sequence converted 1,549,924 raw LIS rows into a longitudinal CKD prediction cohort without using future outcomes. Kidney-related filters retained 1,549,903 rows, typed linkage identified 13,207 patients, and objective same-domain persistence confirmed CKD in 1,702. After post-confirmation, pre-outcome and index-eligibility gates, 637 patients contributed 2,528 indices (Figure 1). Development included 386 patients. A further 251 previously unseen patients contributed later-period indices; 144 patients already represented in development were removed before primary temporal evaluation. Patient-level characteristics at the first eligible index are summarized in Table 1. The 2,528 indices include 502 later-period indices from 144 patients already represented in development; these indices were excluded from the primary temporal evaluation. The primary split denominators before horizon evaluability were 1,463 indices from 386 development patients and 563 indices from 251 unseen temporal patients.

**Table 1.** Patient-level characteristics at the first eligible index. Values are median (interquartile range) or n (%).

Characteristic	Development	Temporal evaluation
Patients	386	251
Age, years	58 (47–68)	62 (49–72)
Male sex	194 (50.3%)	139 (55.4%)
Current eGFR	52.2 (34.9–89.9)	54.3 (37.9–76.6)
Hemoglobin, g/L	122.0 (105.0–137.0)	123.5 (109.0–140.0)
Albumin, g/L	40.7 (37.7–44.4)	41.1 (38.0–43.4)
Potassium, mmol/L	4.46 (4.17–4.88)	4.36 (4.07–4.80)
Phosphate, mmol/L	1.22 (1.08–1.37)	1.22 (1.10–1.36)
Urea, mmol/L	8.11 (5.92–11.46)	7.57 (6.00–10.74)

Horizon-specific evaluability produced nested samples with distinct risk sets. Development contained 1,191 12-month indices from 262 patients (113 positive indices; 48 event patients) and 1,086 24-month indices from 241 patients (223 positive indices; 67 event patients). Temporal evaluation contained 364 12-month indices from 143 patients (21 positive indices; 10 event patients) and 247 24-month indices from 87 patients (50 positive indices; 22 event patients). Every 24-month-evaluable index was also evaluable at 12 months (Figures 1 and 3B). Among positive temporal indices, 17 first met a kidney-failure component and four first met sustained decline by 12 months; the corresponding 24-month counts were 30 and 20 (Figure 3C). Patient and index denominators are reported separately; repeated positive indices from one patient are not independent events.

**Figure 3.**
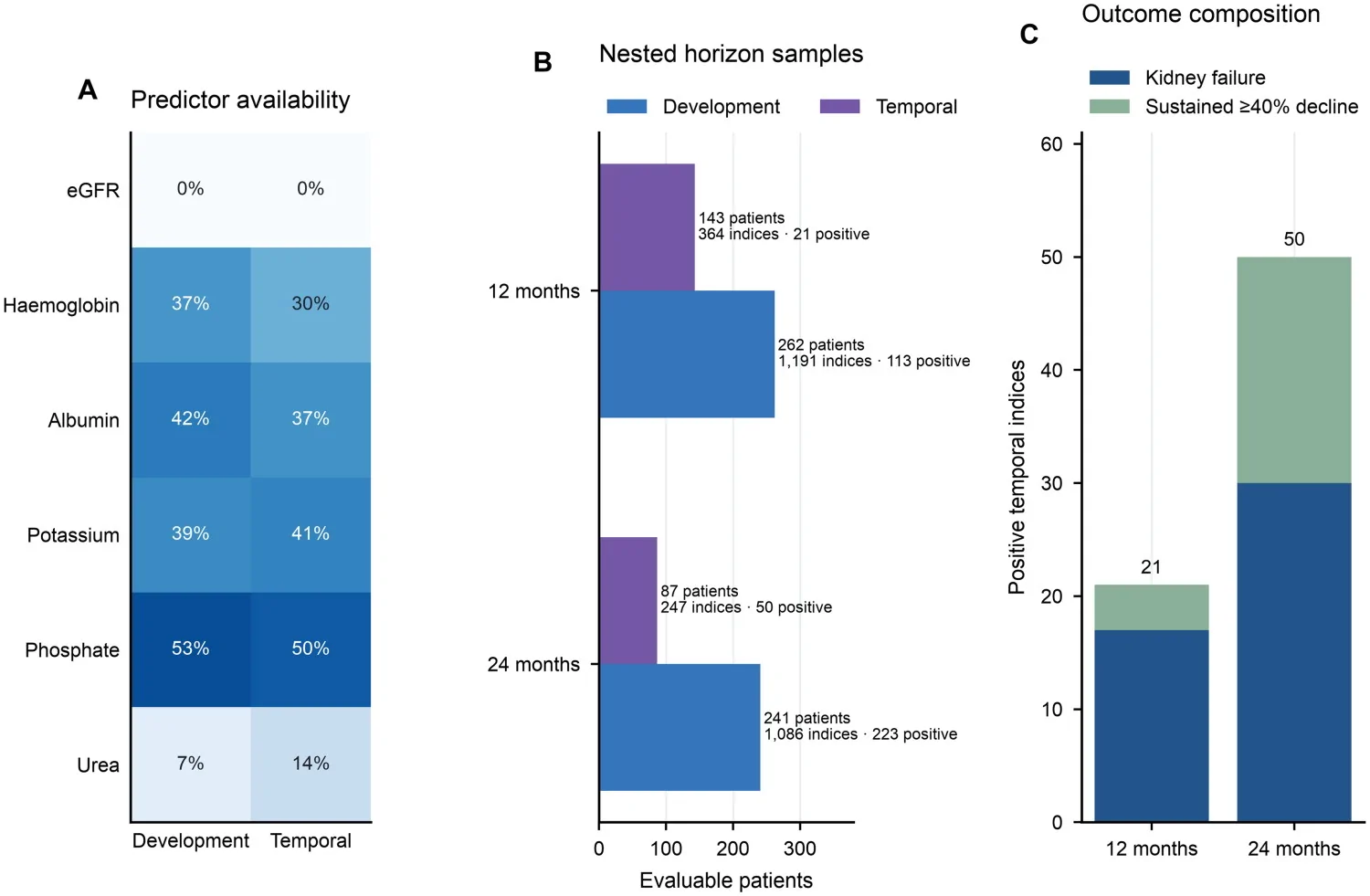
Cohort characteristics and horizon-specific information. Panel A shows missing latest values at each patient’s earliest eligible index. Panel B shows development and temporal patient counts together with the corresponding numbers of prediction indices and positive indices at each horizon. Panel C decomposes the mutually exclusive first temporal outcome components assigned by earliest qualifying date. Independent event-patient counts are 48/67 in development and 10/22 in temporal evaluation at 12/24 months.

At 12 months, 124/386 development patients (32.1%) and 108/251 temporal patients (43.0%) had no evaluable index; the corresponding counts at 24 months were 145/386 (37.6%) and 164/251 (65.3%). Non-evaluable indices numbered 272/1,463 and 199/563 at 12 months, and 377/1,463 and 316/563 at 24 months. Evaluable nonevent indices numbered 1,078 and 343 at 12 months, and 863 and 197 at 24 months. Patients with no positive evaluable index numbered 214 and 133 at 12 months, and 174 and 65 at 24 months. These patient categories do not imply lifetime absence of progression (Supplementary Table S5).

Patient-level characteristics showed that the temporal cohort was modestly older, whereas median current eGFR was similar across periods (Table 1). Availability varied by analyte and calendar period, particularly for phosphate and albumin (Figure 3A). Risk models therefore used fold-fitted imputation with explicit missingness indicators rather than complete-case restriction. In the separate cue module, unavailable defining evidence remained distinct from a negative result.

At the earliest eligible index, development/temporal GFR categories were G1 in 97/39 patients, G2 in 74/66, G3a in 59/56, G3b in 86/52 and G4 in 70/38; G5 indices were excluded. G1–G2 at the later index can coexist with an earlier CKD confirmation based on persistent proteinuria or previously reduced eGFR; current GFR category does not identify the original confirmation pathway. Missingness was 36.5%/29.9% for hemoglobin, 42.5%/37.5% for albumin, 39.1%/40.6% for potassium, 53.1%/50.2% for phosphate and 7.0%/14.3% for urea. Descriptive comparisons by missingness and evaluability are reported in Supplementary Tables S5 and S7–S9; these comparisons do not establish a missing-at-random mechanism.

### One joint grid selected a current-state, task-specific risk pipeline

Selection proceeded within one prespecified development grid rather than through separate rounds of feature and algorithm searching. The four algorithms were first compared for each feature-family–component pair (Figure 4A,B). For 12-month cumulative risk, the within-family choices were elastic net for the core family (AUPRC 0.587), elastic net for the current-state family (0.608), and random forest for the expanded longitudinal family (0.567). For conditional risk during months 13–24, the corresponding choices were elastic net (0.434), random forest (0.404), and random forest (0.383). These six choices came from the same 24-configuration grid and show that algorithm selection was component-specific; no single algorithm was imposed across tasks.

**Figure 4.**
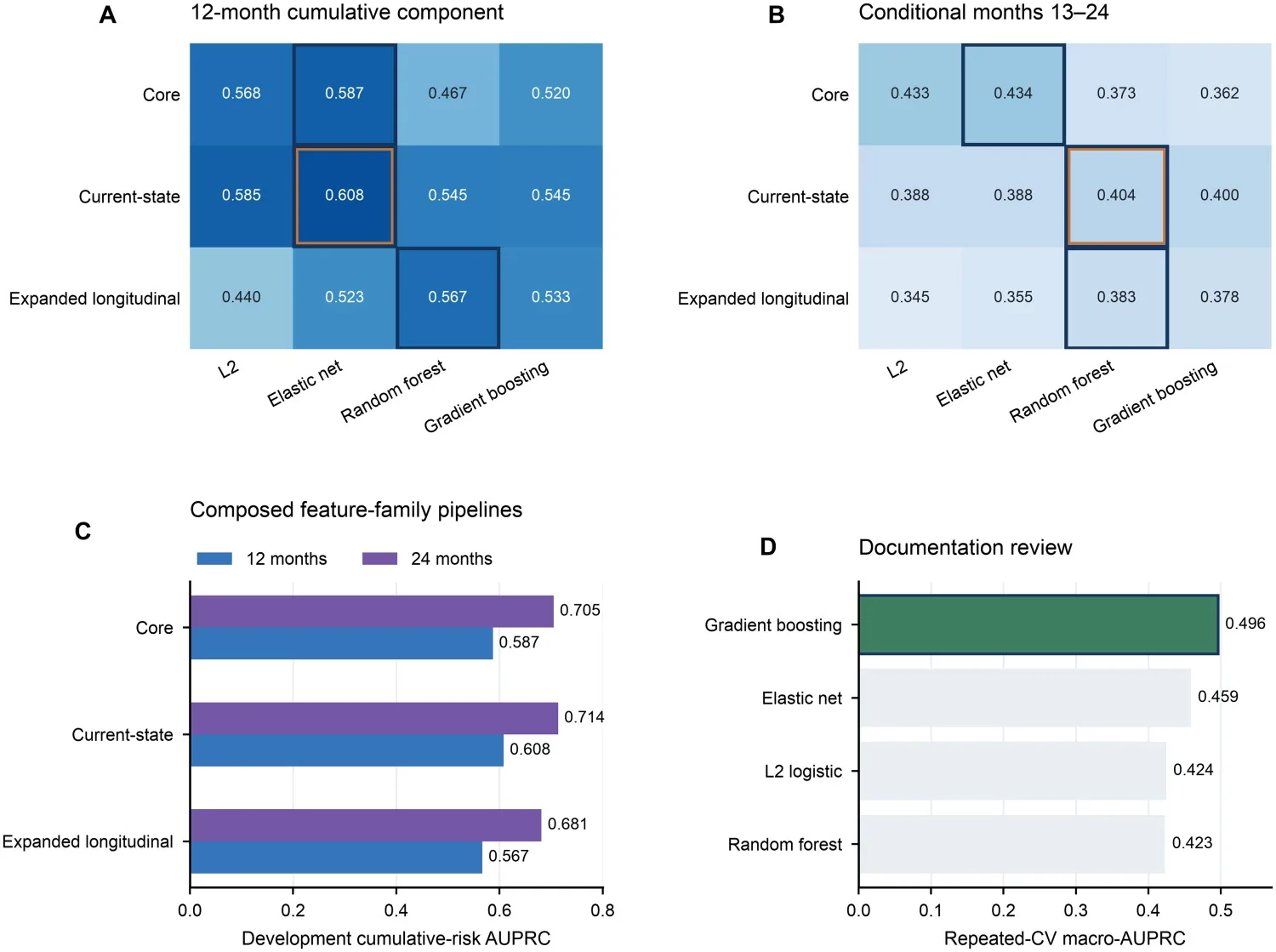
Development-only joint pipeline selection. Panels A and B show the complete repeated patient-grouped cross-validated AUPRC grid for the three feature families and four candidate algorithms at the 12-month cumulative and conditional months 13–24 components. Navy outlines identify the algorithm selected within each family; orange outlines identify the two components of the retained current-state pipeline. Panel C compares the three composed shared-family pipelines at the cumulative 12- and coherent 24-month horizons. Panel D shows the independent macro-AUPRC comparison for documentation-review targets. Complete AUPRC, AUROC and Brier results are reported in Supplementary Table S3 and Supplementary Figure S2.

The selected components were composed within each shared feature family and compared at both cumulative horizons (Figure 4C). Development AUPRCs for the current-state pipeline were 0.608 at 12 months and 0.714 at 24 months, versus 0.587 and 0.705 for the optimized core pipeline and 0.567 and 0.681 for the optimized expanded pipeline. Corresponding Brier scores were 0.065 and 0.107, 0.068 and 0.114, and 0.067 and 0.111, respectively (Supplementary Table S3; Supplementary Figure S2). Thus, within the evaluated grid, the 365-day summaries did not improve cumulative-horizon ranking. The higher isolated conditional AUPRC of the core family did not override the requirement that both components use one feature family.

Documentation-review model selection was independent of the risk grid. Gradient boosting produced the highest development macro-AUPRC among the four cue algorithms (0.496; Figure 4D), with mAP@3 of 0.945 (Table 2). The current-state elastic-net 12-month model, current-state random-forest conditional model, and gradient-boosting review-cue model were therefore frozen before temporal evaluation. In the repeat-level risk-model audit, the elastic-net/random-forest component pair was selected within the current-state family in all five prespecified repeats; coherent 24-month AUPRC ranged from 0.702 to 0.718, AUROC from 0.881 to 0.890 and Brier score from 0.106 to 0.110. Current-state had the highest coherent 24-month AUPRC in 5/5 repeats, whereas its 12-month AUPRC was highest in 3/5 and the core family was highest in 2/5 (Supplementary Table S19). Later-period performance and the synthetic clinical display could not influence any selection decision.

**Table 2.** Development-only locked specifications. The first two rows are the primary risk components selected from the joint candidate grid; the third is the separately selected direct 24-month sensitivity comparator. These rows report development AUPRC, AUROC and Brier score. The documentation-review row was selected independently and reports macro-AUPRC, micro-AUPRC and mAP@3.

Task	Locked model	AUPRC/macro-AUPRC	AUROC/micro-AUPRC	Brier/mAP@3
12-month cumulative	Elastic-net logistic	0.608	0.891	0.065
Conditional months 13–24	Random forest	0.404	0.818	0.105
Direct 24-month sensitivity	L2-penalized logistic	0.705	0.874	0.110
Documentation review	Gradient boosting	0.496 macro	0.622 micro	0.945 mAP@3

### Temporal performance and comparison with the core model

Among 143 temporal patients, including 10 event patients (21 positive and 343 nonevent indices), the development-locked 12-month elastic-net model had the following performance: AUPRC was 0.510 (95% CI 0.217–0.789) against a patient-weighted event prevalence of 0.061, AUROC was 0.937 (0.880–0.981), and Brier score was 0.042 (0.022–0.068). In the same indices, the fixed age-sex-current-eGFR core comparator produced AUPRC 0.519, AUROC 0.927 and Brier score 0.043. Paired differences were −0.009 (−0.171 to 0.123) for AUPRC, 0.010 (−0.020 to 0.045) for AUROC and 0.0003 (−0.0082 to 0.0084) for Brier improvement, defined as core minus locked (Table 3; Figure 5A,C,D).

**Figure 5.**
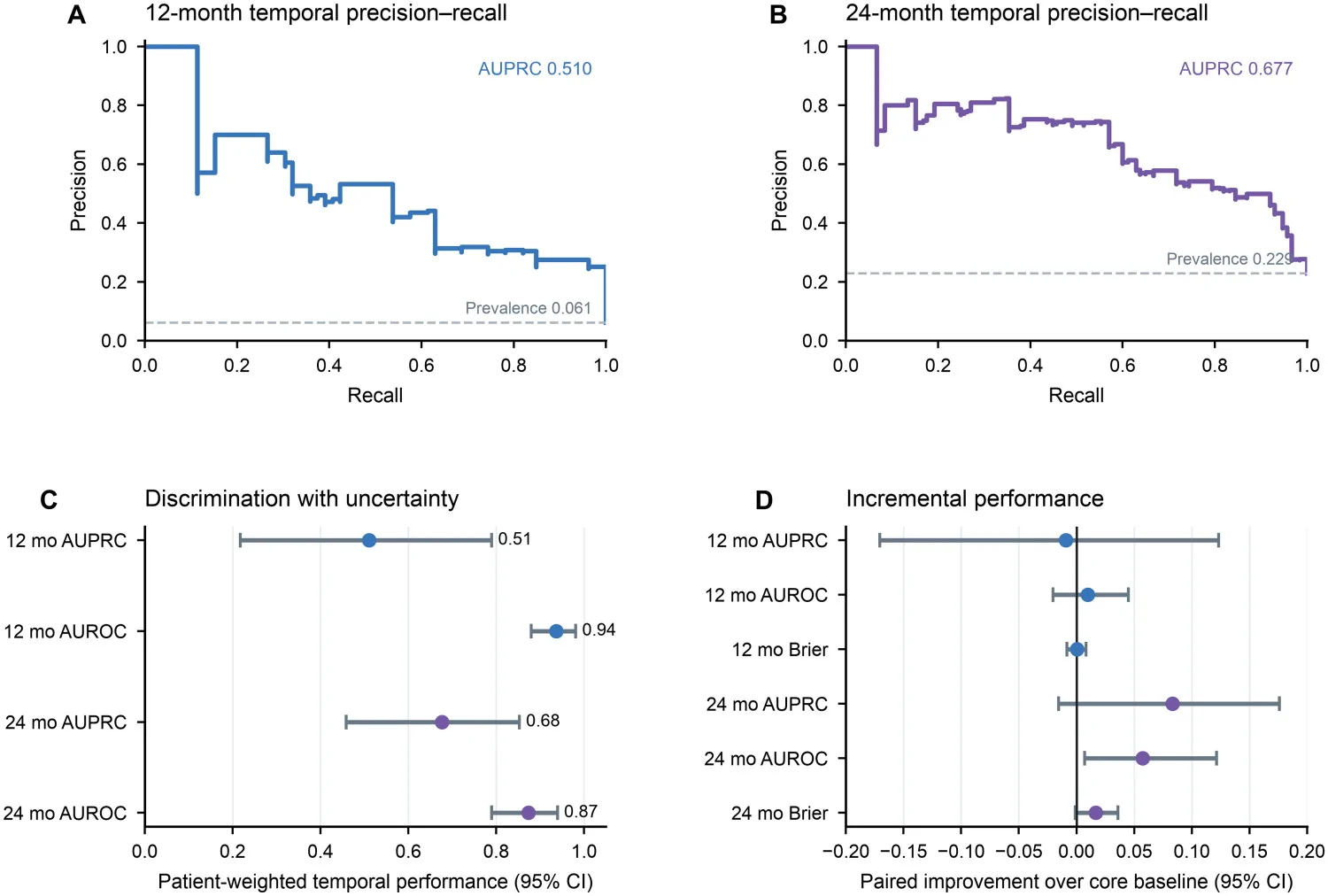
Patient-disjoint temporal performance of the two locked risk outputs. Panels A and B show 12- and 24-month precision–recall curves with patient-weighted prevalence references. Panel C reports absolute AUPRC and AUROC with 95% patient-cluster bootstrap confidence intervals. Panel D reports paired differences from the age-sex-current-eGFR core model; positive values favor the locked model, with Brier expressed as core minus locked. At 12 months, 143 patients include 10 event patients and 21/364 positive indices; at 24 months, 87 patients include 22 event patients and 50/247 positive indices. Wide intervals limit inference.

**Table 3.** Absolute and incremental temporal performance of the locked risk outputs. Core reference predictors were age, sex and current eGFR. Positive paired differences favor the locked model; brier improvement is core minus locked. Confidence intervals use 2,000 patient-cluster bootstrap samples. At 12 months: 143 patients, 10 event patients, 21 positive/343 nonevent indices. At 24 months: 87 patients, 22 event patients, 50 positive/197 nonevent indices. Incremental 12-month performance remained uncertain because all paired intervals included zero and the event count was small.

Horizon	Metric	Locked model (95% CI)	Core reference	Paired improvement (95% CI)
12 months	AUPRC	0.510 (0.217–0.789)	0.519	−0.009 (−0.171 to 0.123)
12 months	AUROC	0.937 (0.880–0.981)	0.927	0.010 (−0.020 to 0.045)
12 months	Brier score	0.042 (0.022–0.068)	0.043	0.0003 (−0.0082 to 0.0084)
24 months	AUPRC	0.677 (0.459–0.853)	0.593	0.083 (−0.016 to 0.176)
24 months	AUROC	0.873 (0.790–0.940)	0.816	0.057 (0.007–0.121)
24 months	Brier score	0.116 (0.076–0.159)	0.133	0.017 (−0.001 to 0.036)

All three paired confidence intervals included zero (Table 3; Figure 5C,D). With only 10 event patients, incremental performance at 12 months could not be conclusively determined; neither superiority, inferiority nor equivalence was established. The locked specification was retained to preserve the development-to-temporal evaluation boundary, not because temporal superiority had been demonstrated.

Among 87 temporal patients, including 22 event patients (50 positive and 197 nonevent indices), coherent 24-month performance was: AUPRC was 0.677 (95% CI 0.459–0.853) against a patient-weighted event prevalence of 0.229, AUROC was 0.873 (0.790–0.940), and Brier score was 0.116 (0.076–0.159). The core comparator produced AUPRC 0.593, AUROC 0.816 and Brier score 0.133. Paired improvement favored the coherent output by 0.083 (−0.016 to 0.176) for AUPRC, 0.057 (0.007–0.121) for AUROC and 0.017 (−0.001 to 0.036) for Brier score (Table 3; Figure 5B–D).

Only the 24-month AUROC difference excluded zero, supporting improved discrimination relative to the core reference. This isolated discrimination result does not establish overall model superiority or clinical benefit. Clinical utility was not evaluated. The AUPRC and Brier differences remained imprecise. Calibration-in-the-large was −0.151 (95% CI −1.189 to 0.596) at 12 months and 0.273 (−0.316 to 0.765) at 24 months; calibration slopes were 0.996 (0.689–1.640) and 1.028 (0.676–1.661). These wide intervals do not confirm reliable individual-risk calibration. Supplementary Table S11 reports joint intercepts, conditional and direct-model calibration, Brier scores and quartile error; Supplementary Figure S3 shows descriptive curves for the 12-month, coherent 24-month, direct 24-month and conditional 13–24-month predictions. These analyses assessed calibration; no recalibration was applied.

In the original absolute-performance bootstrap draws, the median number of distinct event patients represented was 6 at 12 months (central 95% range 3–9) and 14 at 24 months (10–18), with median event-patient draw counts of 10 and 22 when repeated selections were counted. No replicate lacked events. Resampling quantifies uncertainty conditional on this cohort and cannot create additional independent event information (Supplementary Table S12). The exploratory clinical-history proxy comparator yielded AUPRC 0.419/0.565, AUROC 0.917/0.805 and Brier score 0.047/0.136 at 12/24 months; these descriptive comparisons do not establish superiority over established clinical tools (Supplementary Table S16).

The fixed published Tangri three-variable score yielded temporal AUPRC/AUROC of 0.519/0.927 at 12 months and 0.603/0.820 at 24 months, compared with 0.510/0.937 and 0.677/0.873 for the locked model on the same indices. Locked-minus-score paired differences were −0.008 (95% CI −0.179 to 0.112) and 0.010 (−0.019 to 0.046) at 12 months, and 0.074 (−0.025 to 0.178) and 0.053 (0.003 to 0.119) at 24 months, for AUPRC and AUROC, respectively. Target-range sensitivity results were similar. These are exploratory ranking comparisons against a fixed published linear predictor under endpoint and population mismatch; they are not calibration validation of KFRE and do not establish superiority (Supplementary Table S18).

### Horizon coherence and comparison with direct 24-month modeling

A separately fitted cumulative 24-month model did not ensure ordered predictions: seven development indices had a lower 24-month than cross-fitted 12-month estimate, whereas conditional composition eliminated reversals by construction (Figure 6C). In temporal evaluation, AUPRC was 0.677 for the coherent output and 0.672 for the direct comparator. Paired differences were 0.005 (95% CI −0.068 to 0.066) for AUPRC, 0.009 (−0.035 to 0.055) for AUROC and 0.005 (−0.009 to 0.020) for Brier improvement. Positive values favored the coherent output: AUPRC and AUROC differences were coherent minus direct, whereas Brier improvement was direct minus coherent (Figure 6D). All intervals included zero. Horizon coherence is therefore a structural property; a performance advantage or non-inferiority was not established. Composition increased 146/247 probabilities and decreased 101/247, raising the patient-weighted mean from 0.193 to 0.199; relative to p12, it can only increase or remain unchanged. Conditional-model calibration among 80 patients, including 15 with positive conditional indices, is reported in Supplementary Table S11.

**Figure 6.**
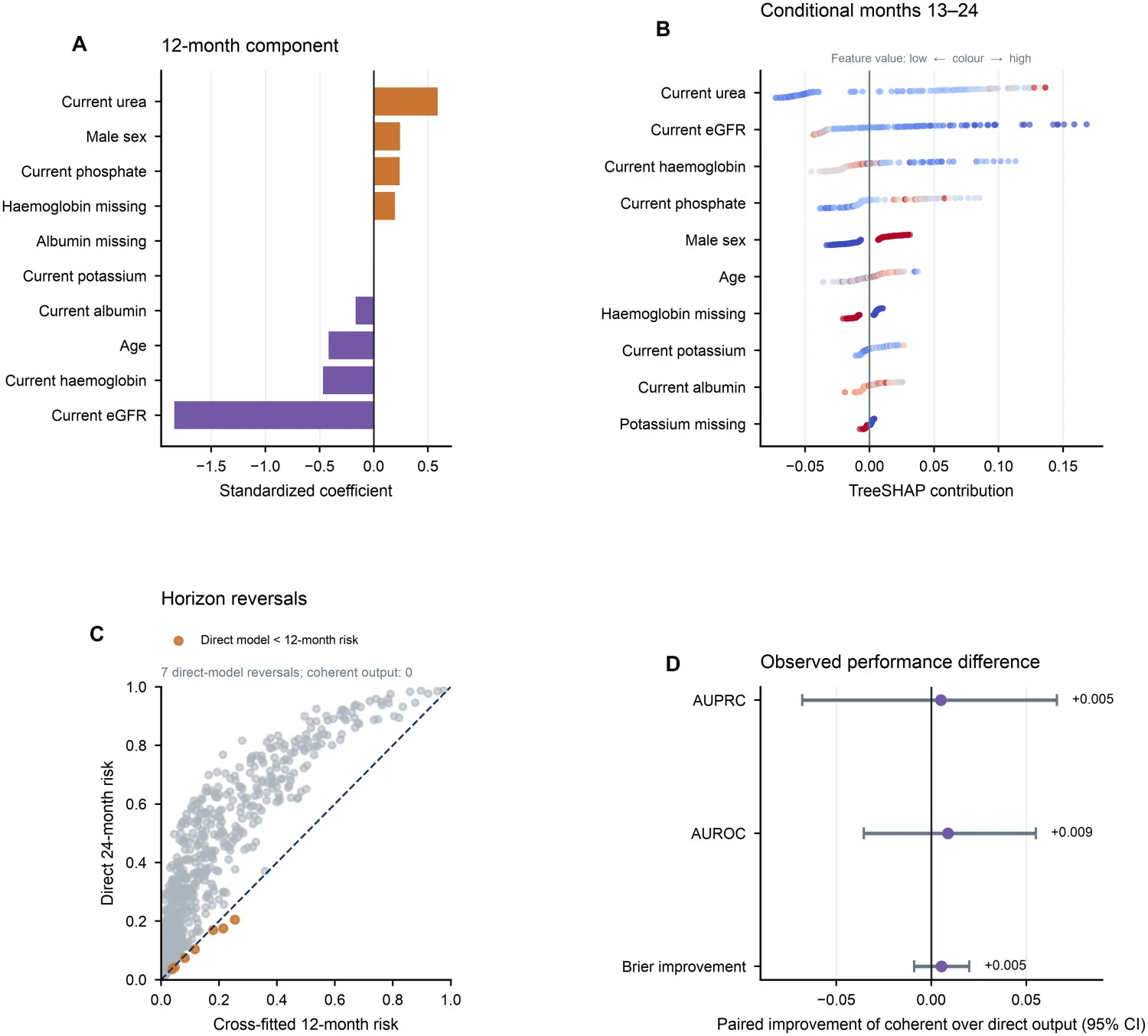
Model explanations and horizon coherence. Panel A shows standardized coefficients for the selected 12-month elastic-net model. Panel B shows TreeSHAP contributions for the selected conditional months 13–24 random forest. Panel C identifies direct-model horizon reversals against cross-fitted 12-month risk. Panel D reports paired temporal differences between coherent and direct 24-month outputs; positive values favor the coherent output, with Brier expressed as direct minus coherent. Coherence is an algebraic property; no predictive or clinical advantage over the direct model is established.

The fitted models assigned prominent contributions to current eGFR and urea, with hemoglobin, phosphate, age and measurement availability contributing according to the risk component and model family (Figure 6A,B). These explanations clarify how the locked outputs were formed but describe fitted associations rather than causal or treatment effects.

### Documentation ranking reflected local recording patterns rather than disease diagnosis

The five laboratory rules produced descriptive positive, negative or not-evaluable states, without diagnostic-performance testing (Figure 7A). The documentation model had macro-AUPRC 0.433 (95% CI 0.301–0.657) and micro-AUPRC 0.480 (0.294–0.663). Among 50 positive-target indices from 30 patients and only three temporally estimable labels, mAP@1 was 0.765, mAP@2 0.863 and mAP@3 0.876 (0.789–0.957). Recall@3 was 1 by construction because all three evaluated labels were returned. It should not be interpreted as evidence of clinical or diagnostic sensitivity (Figure 7B; Supplementary Table S14).

**Figure 7.**
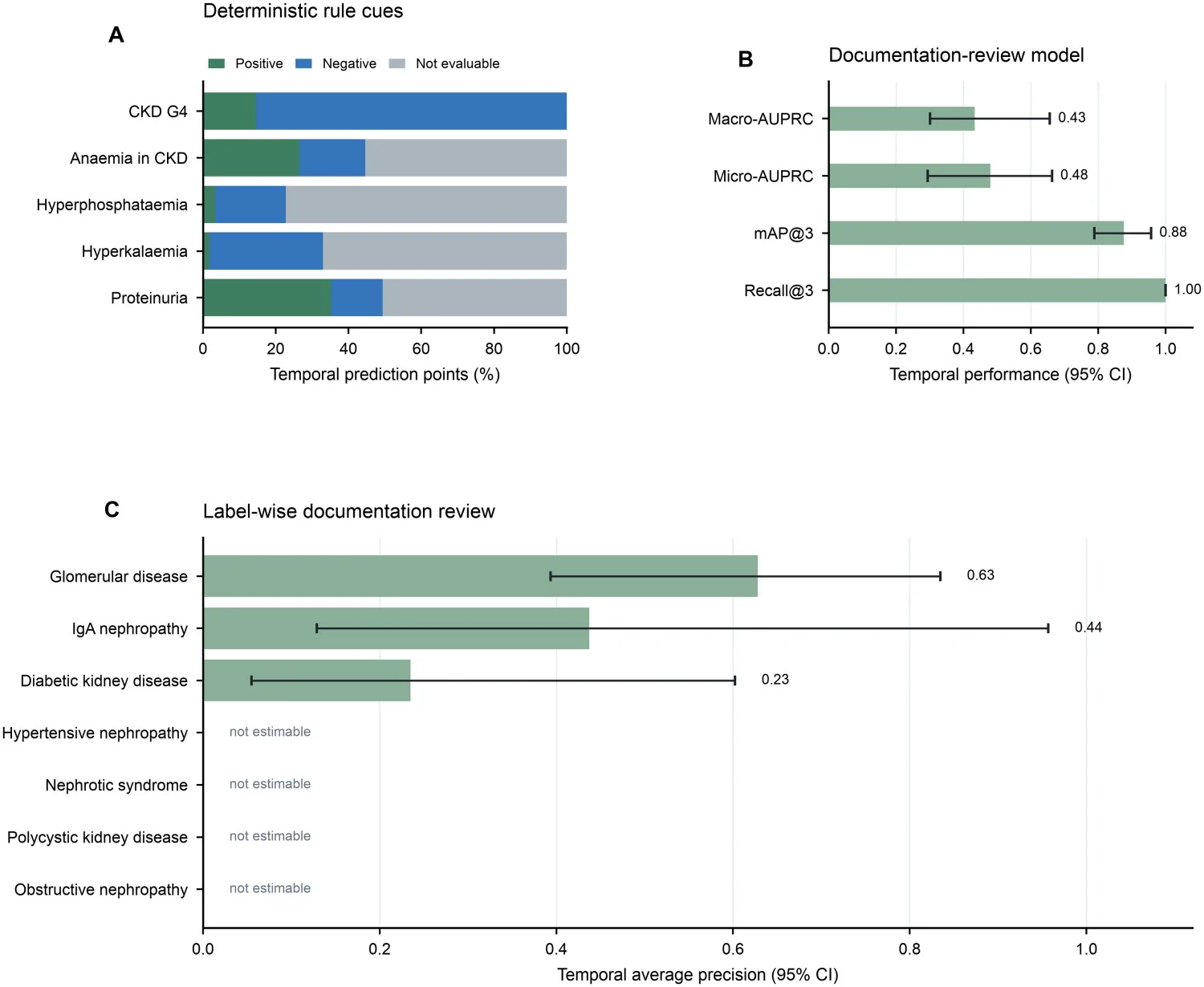
Temporal performance and information boundary of same-day review cues. Panel A shows the three-state distribution of deterministic laboratory cues. Panel B reports overall documentation-ranking metrics with 95% patient-cluster bootstrap confidence intervals. Panel C reports label-wise average precision and explicitly suppresses labels that failed the prespecified estimability rule. Ranking uses only three temporally estimable labels; recall@3 = 1 is guaranteed. Positive-target ranking includes 50 indices from 30 patients. Prior-history comparator results are in Supplementary Table S14. Documentation-review outputs refer to recorded mentions, not adjudicated disease etiology.

Label-wise average precision was 0.628 for glomerular disease mention (19 positive patients), 0.437 for IgA nephropathy mention (5) and 0.235 for diabetic kidney disease mention (6). Four other labels failed the temporal estimability rule (Table 4; Figure 7C). In the simple-baseline comparison, mAP@3 was 0.784 for development-prevalence ranking, 0.871 for any prior mention and 0.915 for prior-year mention counts. Model-minus-comparator differences were 0.093 (95% CI 0.039–0.158), 0.005 (−0.053 to 0.065) and −0.039 (−0.114 to 0.038), respectively. The fitted model improved on prevalence ranking but did not establish additional ranking value over documentation history; a simple count-based ranking had a higher point estimate (Supplementary Table S14).

**Table 4.** Label-wise temporal performance of the locked weakly supervised cue model. Confidence intervals are shown only for labels meeting the estimability rule.

Documentation-review target	Positive temporal patients	Average precision (95% CI)
Diabetic kidney disease mention	6	0.235 (0.055–0.602)
Hypertensive nephropathy mention	2	Not estimable
Glomerular disease mention	19	0.628 (0.393–0.835)
IgA nephropathy mention	5	0.437 (0.129–0.957)
Nephrotic syndrome mention	2	Not estimable
Polycystic kidney disease mention	4	Not estimable
Obstructive nephropathy mention	0	Not estimable

### Sensitivity analyses showed dependence on the prediction and observation definitions

Sensitivity analyses that altered the temporal split or eGFR definition produced different performance estimates (Figure 8A). With development indices ending on 31 December 2020 and evaluation indices from 2023, AUPRC was 0.591 at 12 months and 0.654 at 24 months. For the uniform-eGFR sensitivity analysis, cohort construction, outcome ascertainment, predictor generation, and model selection and fitting were repeated using the 2021 CKD-EPI equation for all eligible eGFR values. This analysis included 636 patients contributing 2,526 indices; temporal AUPRC was 0.551 at 12 months and 0.706 at 24 months. The same algorithm families were selected as in the primary analysis, but this does not establish resampling stability or remove concern about residual calendar-related measurement heterogeneity. At indices with paired values, GFR-category disagreement occurred in 1/14 development and 43/368 temporal indices (Supplementary Table S10).

**Figure 8.**
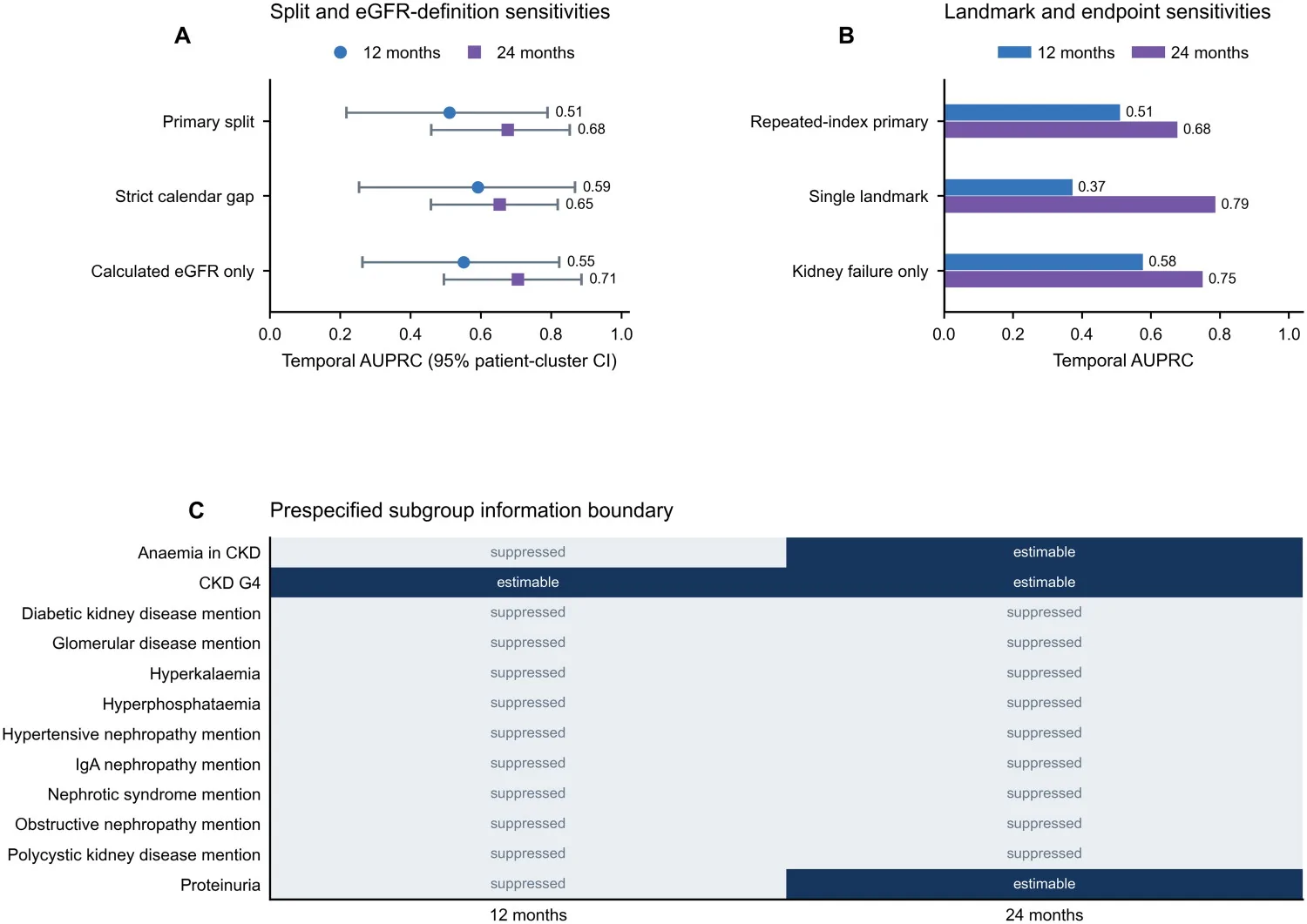
Sensitivity and prespecified subgroup estimability analyses. Panel A compares the primary temporal split with the stricter calendar-gap split and the complete pipeline using uniformly calculated 2021 CKD-EPI eGFR. Panel B reports repeated-index, single-landmark and kidney-failure-only temporal AUPRCs. Panel C identifies cue-positive subgroups meeting the minimum event and nonevent requirement; unsupported estimates are suppressed. Primary event-patient counts are 10/22 at 12/24 months; earliest-landmark counts are 7/19. These analyses change the prediction problem and do not prove uniform robustness.

Changing the prediction occasion materially changed performance (Figure 8B). Selecting one earliest horizon-evaluable index per patient reduced 12-month AUPRC from 0.510 to 0.372, with 7 rather than 10 event patients; at 24 months, AUPRC changed from 0.677 to 0.787, with 19 rather than 22 event patients. The two analyses estimate different encounter-level prediction problems and have different prevalences. In the repeated-index temporal sample, 3/21 positive 12-month indices were within 90 days of confirmed progression and 9/21 within 180 days; later assessments can be more proximal to progression even under equal patient weighting. Kidney-failure-only AUPRCs were 0.577 and 0.750. These results indicate definition-dependent performance, not uniform robustness (Supplementary Table S6).

Removing missingness indicators while retaining median imputation and the locked model specifications yielded AUPRC 0.496/0.679 and Brier score 0.042/0.116 at 12/24 months. Complete-case refitting yielded AUPRC 0.496/0.577 and Brier score 0.048/0.154, but retained only 64/40 temporal patients with 5/12 event patients. These exploratory comparisons cannot disentangle the missing-data strategy from complete-case selection (Supplementary Tables S13 and S15). Reliable UACR measurements within the preceding year were available at only 9/364 12-month temporal indices (5 patients) and 8/247 24-month indices (4 patients), and at no development risk indices. These data could not support a representative comparison with UACR-dependent KFRE versions (Supplementary Table S17). The UACR-independent published three-variable Tangri score was therefore evaluated separately as a ranking comparator, with its endpoint and absolute-risk limitations stated explicitly (Supplementary Table S18).

The subgroup information boundary was assessed separately at each horizon (Figure 8C). CKD G4 met the event/nonevent requirement at both horizons; anemia and proteinuria met it only at 24 months. Other cells failed the requirement. The figure reports eligibility for subgroup performance estimation, not discrimination estimates or evidence of transportability.

### The outputs were combined in a conceptual display

Figure 9 shows a possible way to display the outputs together; it is not a validated clinical decision-support tool. Its wholly synthetic values are not predictions for a real patient and contribute to no performance estimate. The figure distinguishes future progression estimates from measured laboratory states and from documentation-review priorities. External calibration, action thresholds, usability, workflow and clinical impact remain untested.

**Figure 9.**
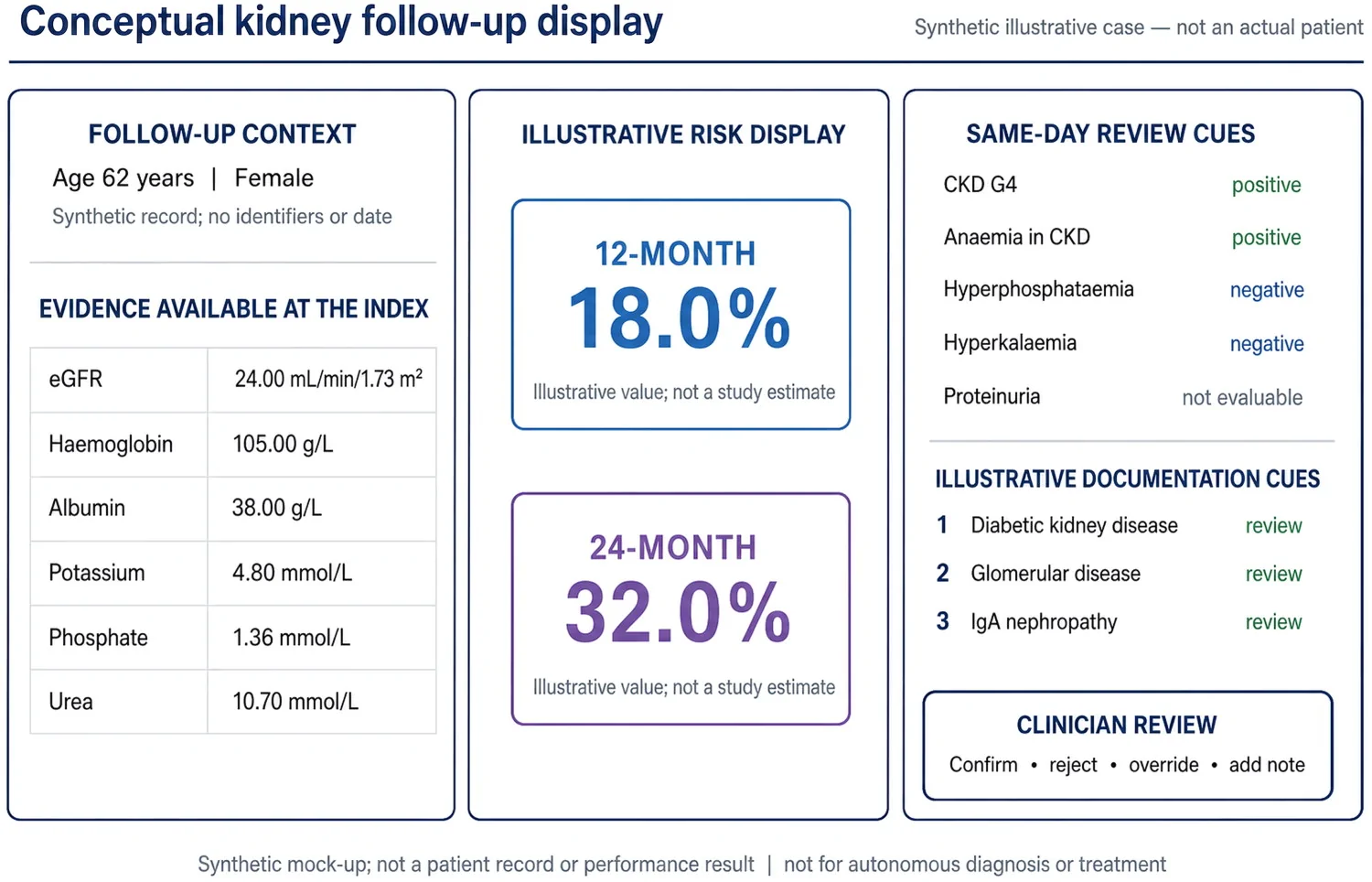
Conceptual kidney follow-up display for research illustration only; not clinically validated. The demographic characteristics, laboratory values, risk scores and documentation ranking are synthetic, not an actual patient or an additional model-performance result. Laboratory states follow the specified rules; illustrative risk values obey horizon ordering. The display has no validated action threshold, prospective workflow evaluation, usability testing or impact assessment and must not guide autonomous diagnosis or treatment.

## Discussion

This study asked whether routine laboratory information available at CKD follow-up could predict documented progression within 12 and 24 months in previously unseen patients from a later period at the same hospital. The 12-month model had an AUROC of 0.937, but only 10 event patients and wide uncertainty precluded an established increment over the core reference. At 24 months, the AUROC difference favored the locked model, whereas AUPRC and Brier differences remained uncertain; this does not establish overall model superiority or clinical benefit. Although calibration-slope point estimates were near their ideal value, wide confidence intervals precluded reliable individual-risk calibration, and no recalibration was performed [15]. The secondary documentation analysis identified local recording patterns without establishing additional value over simple prior-history ranking. Integration of these outputs is conceptual; clinical utility was not evaluated [16].

Repeated measurements can update kidney-failure prediction, and machine-learning models have been evaluated for CKD progression [8,19]. The specified 365-day summaries did not improve the selected pipeline, but this does not imply that longitudinal information is generally unhelpful. Event scarcity, irregular testing, limited trajectory representations and algorithm choice may have contributed; we did not evaluate other window lengths, flexible trajectories or a full observation-intensity model. With only 48 and 67 development event patients at 12 and 24 months, selection may remain sample-dependent. The repeat-level audit supported the current-state component choices and 24-month family ranking, but 12-month family selection was less consistent. Because all repeats reused the same development sample, this consistency does not remove selection optimism or substitute for nested resampling and external replication (Supplementary Table S19).

The 24-month increment represents horizon-specific discrimination against an empirical comparator. The clinical core is not equivalent to established CKD prognostic tools, and the added diagnosis-history and proteinuria proxies are incomplete records rather than adjudicated clinical histories. Four- and eight-variable KFREs could not be evaluated because albuminuria was sparse. We therefore assessed Tangri et al.’s published three-variable age-sex-eGFR linear predictor [5]. Ranking performance was similar at 12 months, while the locked model had higher 24-month point estimates, although uncertainty was material and endpoints, horizons and populations were not fully aligned. Without absolute-risk calibration, this is not a full KFRE validation and does not establish superiority to, or replacement of, established equations (Supplementary Table S18).

Documentation targets reflect what clinicians recorded, not adjudicated causes of CKD [20]. Specialty, encounter context, local language, coding conventions and prior documentation can all influence these labels. Only three of seven targets were temporally estimable, and selecting their Top-3 guarantees complete recall within the evaluated subset. The simple-baseline analysis further limits the claim: the fitted model did not establish improvement over any prior mention or prior-year mention counts. The documentation component should therefore remain exploratory; the current evidence does not justify adding machine-learning complexity to clinical documentation review.

The study assesses prediction; clinical utility was not evaluated. No action threshold, intervention consequence or net-benefit analysis was specified [16]. Potential future applications include prioritizing follow-up, laboratory monitoring, nephrology assessment or structured record review, but none was tested here. Before implementation, independent sites should evaluate frozen models, calibration and comparison with established tools using a harmonized endpoint and measured albuminuria [3,5–7]. Prospective studies should then prespecify actions and thresholds and evaluate safety, workflow burden, usability and patient outcomes. Patient-disjoint temporal evaluation at one institution is not geographic external validation.

The main design strengths are objective CKD confirmation, report-time predictor boundaries, patient-grouped resampling and development-only model selection, consistent with transparent reporting of prediction studies using routinely collected data [9,10]. These safeguards address information leakage, but do not make repeated indices independent. Repeated prediction estimates average performance over a patient’s retained follow-up encounters, whereas a single landmark estimates prediction at the earliest qualifying occasion. The 12-month AUPRC difference between these designs, and the smaller event count in the single-landmark analysis, show why neither should be portrayed as interchangeable evidence of robustness. A 30-day interval may represent a new laboratory assessment without necessarily being a distinct treatment decision.

Informative observation is a central limitation because electronic health records reflect both patient health and interactions with the healthcare system [21]. Temporal evaluation excluded all indices for 43.0% of initially eligible patients at 12 months and 65.3% at 24 months because no horizon-evaluable index was available. Retention depends on returning for sufficiently late kidney-function testing or having a recorded event; observed and unobserved patients may differ in illness severity, treatment, access and continuity of care. Descriptive baseline comparisons cannot exclude this selection bias. More frequent testing also increases opportunities to confirm sustained decline, while progression, dialysis, transplantation or death outside the hospital may be missed. The endpoint is therefore documented in-system progression, and reported risks are conditional on observation. We did not fit a new survival model because reliable death linkage and an adequately supported censoring/observation model were unavailable; ordinary non-informative censoring assumptions would not by themselves solve this problem.

Missingness is also likely to reflect clinical testing decisions. Median imputation with indicators is an operational prediction strategy, not proof that values are missing completely at random or missing at random; missing-not-at-random mechanisms remain plausible. Neither descriptive missingness associations nor complete-case sensitivity can identify the mechanism from observed data alone [22]. The complete-case sample is smaller and selected, and the sensitivity results do not establish robustness to all imputation assumptions. Changes in assay availability, reported eGFR, referral patterns and documentation may further limit transportability. Detailed treatment exposure, cardiovascular history, encounter mix and nephrology follow-up were not available as validated clinical covariates in the analytical dataset.

Precision remains limited by 10 and 22 temporal event patients. Calibration measures are therefore reported with cluster confidence intervals rather than treated as established; subgroup estimates are less precise. Earlier rules of thumb proposed at least 100 events and 100 nonevents for external validation, whereas Riley et al. recommend model- and precision-specific calculations [23]. In an illustrative one-index-per-patient calculation, the chosen precision targets required 7,470 patients (approximately 366 events) for the 12-month model and 2,381 (approximately 520 events) for the 24-month model; observed-to-expected (O/E) ratio and calibration-slope precision were the respective binding criteria (Supplementary Table S20). These scenario-dependent targets are neither retrospective power calculations nor universal requirements. A future multisite protocol should update them for its population, outcome prevalence, prediction-time definition and precision targets; repeated indices cannot replace independent event patients.

The laboratory review rules have a different evidential role. For example, phosphate has established physiological and pathological relevance to mineral homeostasis, supporting attention to measured hyperphosphatemia [24]. This biological rationale does not validate our chosen cue threshold as a treatment rule or demonstrate a clinical benefit from displaying it.

In conclusion, this study evaluated 12- and coherent 24-month prediction of documented CKD progression in previously unseen patients from a later period at one institution. The estimates apply to horizon-evaluable patients and do not represent unconditional risk among all patients presenting for CKD follow-up. Incremental performance was horizon-dependent: uncertainty remained at 12 months, whereas 24-month AUROC was higher than that of the age-sex-current-eGFR core comparator; AUPRC and Brier differences remained uncertain. Clinical utility was not evaluated. Calibration and transportability remain uncertain [15], and the exploratory documentation module did not establish improvement over simple history-based ranking. Multisite external validation with adequate independent event counts, established comparators [3,5–7] and prospective evaluation of clinical use [16] are essential before implementation.

## Supplementary Material

Renal_Failure_Supplementary_File_1.docx

## Data Availability

The reproducibility materials supporting this study are deposited in Zenodo [25] (https://doi.org/10.5281/zenodo.23039194). The publicly accessible record contains restricted files, including the analysis code, exact documentation-label rules, frozen model specifications, synthetic example data and non-identifying aggregate outputs. Qualified researchers and journal editors may request access by signing in to Zenodo and selecting ‘Request access’ on the record page; requests should state the purpose of access and are reviewed by the record owners. Patient-level source data are not deposited because they contain sensitive longitudinal health information and potentially identifying dates. Requests from qualified researchers for a minimum de-identified dataset remain subject to review by the institutional ethics committee and data custodian, applicable ethics approval and execution of a data-use agreement.
