# Supplementary material for: Predicting 12- and 24-month documented CKD progression from routine laboratory data: a single-center temporal evaluation
Source: Ren Fail. 2026 Oct 28;48(1):2753249. doi: 10.1080/0886022X.2026.2753249 (PMC13717766; doi:10.1080/0886022X.2026.2753249)
Supplement: Supplementary file 1 [file IRNF_A_2753249_SM5976.docx]

Supplementary File 1 — Supplementary Material

Predicting 12- and 24-month documented CKD progression from routine laboratory data: a single-centre temporal evaluation

Mudan Jin†; Jie Jing†; Di Lin†; Jing Li; Lang Zhang; Pinhao Guo; Zheng Wang*; Xingzhu Wen*

# Supplementary methods

All analyses retain typed identity linkage, report-time boundaries, objective CKD confirmation, patient weighting and the patient-disjoint temporal split. Laboratory cues require index-date collection and reporting by the index time; missing valid evidence is not evaluable. Additional analyses are exploratory and did not select or recalibrate the frozen primary model.

Risk-model selection used one development-only joint grid: three feature families × four algorithms × two risk components. Algorithms were chosen within each family and component from five repeats of patient-grouped five-fold cross-validation using the specified AUPRC, Brier and simplicity rule. Component pairs formed coherent shared-family pipelines. The expanded longitudinal family could replace the current-state family only by improving AUPRC without worsening Brier score. No second search or temporal reselection was performed. A repeat-level audit reapplied this rule separately within each of the five original repeats; it did not change the locked models; the repeat-level results are reported below.

The uniform-eGFR sensitivity repeated cohort construction, outcome ascertainment, predictor generation, and model selection and fitting using the 2021 CKD-EPI equation for every eligible eGFR value. Reported eGFR remained preferred in the frozen primary analysis.

Additional analyses quantify horizon evaluability and selection, index frequency and event proximity, case mix and missingness, eGFR source and paired agreement, calibration, bootstrap event information, complete-case evaluation of frozen scores, simple documentation-ranking comparators, fixed-specification imputation sensitivities, a clinical-history proxy comparator, usable urine albumin-to-creatinine ratio availability, a published three-variable Tangri score comparison, repeat-level model-selection stability, and precision-based external-validation planning. Seeds, analysis code, exact documentation-label rules, frozen model specifications and aggregate results are provided in the restricted-access Zenodo reproducibility package (DOI: 10.5281/zenodo.23039194). Qualified researchers and journal editors may request access by signing in to Zenodo and selecting “Request access” on the record page.

## Supplementary Table S1. Laboratory and documentation-review definitions.

| **Cue** | **Positive definition** | **Evaluability rule** |
| --- | --- | --- |
| CKD G4 | Index eGFR 15–29.9 mL/min/1.73 m² | Always evaluable when the risk index is eligible |
| Anaemia in CKD | Index-date haemoglobin <130 g/L (men) or <120 g/L (women) | Not evaluable without valid index-date haemoglobin |
| Hyperphosphataemia | Index-date phosphate >1.45 mmol/L | Not evaluable without valid index-date phosphate |
| Hyperkalaemia | Index-date potassium >5.5 mmol/L | Not evaluable without valid index-date potassium |
| Proteinuria | Dipstick ≥1+ or 24-hour protein ≥150 mg/day (≥0.15 g/day) | Not evaluable without a qualifying urine assay |
| Documentation-review targets | Seven prespecified index-date patterns; exact rules in the restricted-access Zenodo reproducibility package (DOI: 10.5281/zenodo.23039194), documentation/Documentation_Label_Rules.md | Absence means no documentation mention, not absence of disease |

## Supplementary Table S2. Frozen candidate-model specifications.

| **Candidate and task** | **Frozen implementation** |
| --- | --- |
| All tasks: L2 logistic | Median imputation with indicators; standardization; C=0.2; liblinear |
| All tasks: elastic-net logistic | Median imputation with indicators; standardization; C=0.2; l1 ratio=0.5; saga |
| Risk: random forest | Median imputation with indicators; 800 trees; maximum depth 5; minimum leaf 15; sqrt feature sampling |
| Cue: random forest | Median imputation with indicators; 500 trees; maximum depth 5; minimum leaf 15; sqrt feature sampling |
| Risk: gradient boosting | Median imputation with indicators; 150 iterations; 8 leaves; minimum leaf 20; learning rate 0.04; L2=5 |
| Cue: gradient boosting | Median imputation with indicators; 120 iterations; 8 leaves; minimum leaf 20; learning rate 0.04; L2=5 |

## Supplementary Table S3. Development feature-family pipeline benchmark.

| **Family** | **12m AP** | **12m AUC** | **12m Brier** | **24m AP** | **24m AUC** | **24m Brier** |
| --- | --- | --- | --- | --- | --- | --- |
| Core | 0.587 | 0.903 | 0.068 | 0.705 | 0.863 | 0.114 |
| Current state | 0.608 | 0.891 | 0.065 | 0.714 | 0.888 | 0.107 |
| Expanded 365-day | 0.567 | 0.901 | 0.067 | 0.681 | 0.886 | 0.111 |

AP denotes average precision, reported as AUPRC; AUC denotes AUROC. These are repeated grouped out-of-fold estimates used during selection, not nested selection-adjusted validation. Conditional-component development AUPRC/AUROC/Brier were 0.404/0.818/0.105. The complete 24-candidate grid is shown later in this file and in the reproducibility package.

## Supplementary Fig. S1. Assay quality-control retention and missing latest current-state values at each patient’s earliest eligible index. Panel A shows retained assay rows and rows excluded by quality control. Panel B shows the percentages missing each latest current-state value in the development and temporal cohorts.

## Supplementary Fig. S2. Complete development candidate metrics for the 12-month cumulative and conditional months 13–24 components. The left block reports 12-month AUPRC, AUROC and Brier score; the right block reports the corresponding conditional-component metrics. Rows combine the three feature families with the four prespecified algorithms.

## Supplementary Fig. S3. Patient-weighted temporal calibration by prediction quartile. A: 12-month cumulative risk (143 patients; 10 event patients). B: coherent 24-month risk and C: direct 24-month risk (each 87 patients; 22 event patients). D: conditional months 13–24 (80 patients; 15 event patients, among 24-month-evaluable indices event-free through day 365). Each panel uses its own prediction quartiles. Numeric calibration uncertainty is reported in the tables below. No recalibration was applied.

Calibration error depends on the selected bins. With few independent event patients, these plots and the near-unit slope point estimates do not establish accurate individual probabilities.

## Supplementary Table S4. Methods–results crosswalk. Supplementary Tables S5–S20 follow in numerical order.

| **Result** | **Method** | **Location** |
| --- | --- | --- |
| Cohort and horizons | Eligibility and evaluability | Fig. 1; Tables 1, S5–S10 |
| Selection | Development-only grouped resampling | Fig. 4; Tables 2, S3; Fig. S2 |
| Temporal performance | Frozen predictions, patient weights and cluster bootstrap | Table 3; Fig. 5; Table S12 |
| Calibration | Weighted offset intercept, joint intercept/slope and quartiles | Table S11; Fig. S3 |
| Coherence | Conditional composition versus direct model | Fig. 6; Table S11 |
| Documentation review | Text targets, three estimable labels; simple baselines | Table 4; Fig. 7; Table S14 |
| Sensitivity | Gap, eGFR, landmark, endpoint, missing-data specification | Fig. 8; Tables S13, S15 |
| Clinical context | Clinical-history proxy comparator; UACR availability | Tables S16–S17 |
| Conceptual display | Synthetic example; no clinical validation | Fig. 9 |

## Supplementary Table S5. Horizon evaluability and observation selection.

Totals are before horizon evaluability within each primary split. Patients with no evaluable index are distinct from non-evaluable indices contributed by patients who have another evaluable index. “Non-event patients” below denotes patients with no positive evaluable index, not lifelong absence of an event.

### Development

| **Measure** | **12 months** | **24 months** |
| --- | --- | --- |
| Initially eligible patients | 386 | 386 |
| Initially eligible indices | 1463 | 1463 |
| Evaluable patients | 262 | 241 |
| Evaluable indices | 1191 | 1086 |
| Event patients | 48 | 67 |
| Patients with no positive index | 214 | 174 |
| Positive indices | 113 | 223 |
| Non-event indices | 1078 | 863 |
| Patients with no evaluable index | 124 | 145 |
| Non-evaluable indices | 272 | 377 |

### Temporal evaluation

| **Measure** | **12 months** | **24 months** |
| --- | --- | --- |
| Initially eligible patients | 251 | 251 |
| Initially eligible indices | 563 | 563 |
| Evaluable patients | 143 | 87 |
| Evaluable indices | 364 | 247 |
| Event patients | 10 | 22 |
| Patients with no positive index | 133 | 65 |
| Positive indices | 21 | 50 |
| Non-event indices | 343 | 197 |
| Patients with no evaluable index | 108 | 164 |
| Non-evaluable indices | 199 | 316 |

### Table S5 (continued). Comparison at the earliest eligible index, classified by whether any index was horizon-evaluable:

| **Split / horizon** | **Group** | **Patients** | **Age median** | **eGFR median** | **Male %** | **Prior tests median** |
| --- | --- | --- | --- | --- | --- | --- |
| Dev. / 12m | evaluable | 262 | 58.0 | 48.3 | 50.0 | 2.0 |
| Dev. / 12m | non_evaluable | 124 | 58.5 | 66.0 | 50.8 | 2.0 |
| Dev. / 24m | evaluable | 241 | 58.0 | 49.9 | 49.4 | 2.0 |
| Dev. / 24m | non_evaluable | 145 | 59.0 | 61.4 | 51.7 | 2.0 |
| Temporal / 12m | evaluable | 143 | 60.0 | 55.6 | 55.2 | 2.0 |
| Temporal / 12m | non_evaluable | 108 | 62.5 | 54.2 | 55.6 | 2.0 |
| Temporal / 24m | evaluable | 87 | 62.0 | 52.2 | 57.5 | 2.0 |
| Temporal / 24m | non_evaluable | 164 | 61.5 | 55.6 | 54.3 | 2.0 |

eGFR units: mL/min/1.73 m²; age: years. Prior tests refer to eGFR measurements in the preceding 365 days. Baseline comparability does not establish non-informative follow-up. Additional temporal group distributions are in frozen_aggregate_outputs/postprocess/temporal_evaluability_summary.json.

## Supplementary Table S6. Prediction-index distribution and proximity to confirmed progression.

Repeated indices are correlated occasions, not independent patients. Medians and quartiles describe indices per patient. Event proximity describes positive indices only and is not a causal analysis of testing frequency.

| **Split / sample** | **Patients** | **Indices** | **Median (Q1–Q3)** | **Range** | **One index** |
| --- | --- | --- | --- | --- | --- |
| Dev. / all | 386 | 1463 | 2.0 (1.0–5.0) | 1–37 | 138 |
| Dev. / 12m | 262 | 1191 | 3.0 (2.0–5.0) | 1–37 | 60 |
| Dev. / 24m | 241 | 1086 | 3.0 (2.0–5.0) | 1–36 | 58 |
| Temporal / all | 251 | 563 | 1.0 (1.0–3.0) | 1–12 | 127 |
| Temporal / 12m | 143 | 364 | 2.0 (1.0–3.0) | 1–12 | 65 |
| Temporal / 24m | 87 | 247 | 2.0 (1.0–3.5) | 1–11 | 31 |

| **Split / horizon** | **Positive indices** | **Median days to confirmation** | **≤90 days** | **≤180 days** |
| --- | --- | --- | --- | --- |
| Dev. / 12m | 113 | 241.4 | 6 | 37 |
| Dev. / 24m | 223 | 363.8 | 6 | 37 |
| Temporal / 12m | 21 | 209.4 | 3 | 9 |
| Temporal / 24m | 50 | 396.3 | 3 | 9 |

## Supplementary Table S7. Clinical characterization at the earliest eligible index.

GFR categories describe current eGFR after objective CKD confirmation. Diagnosis-history variables are documentation proxies: an absent mention does not confirm absence of disease. Cardiovascular disease, medications, encounter mix and nephrology follow-up were not adjudicated or available as validated structured covariates.

| **Characteristic** | **Development** | **Temporal** |
| --- | --- | --- |
| G1 | 97/386 (25.1%) | 39/251 (15.5%) |
| G2 | 74/386 (19.2%) | 66/251 (26.3%) |
| G3a | 59/386 (15.3%) | 56/251 (22.3%) |
| G3b | 86/386 (22.3%) | 52/251 (20.7%) |
| G4 | 70/386 (18.1%) | 38/251 (15.1%) |
| G5 | 0/386 (0.0%) | 0/251 (0.0%) |
| Prior diabetes mention | 27/386 (7.0%) | 18/251 (7.2%) |
| Prior hypertension mention | 29/386 (7.5%) | 8/251 (3.2%) |
| Prior kidney-disease mention | 233/386 (60.4%) | 87/251 (34.7%) |
| Index-date proteinuria positive | 120/386 (31.1%) | 96/251 (38.2%) |
| Index-date proteinuria not evaluable | 225/386 (58.3%) | 120/251 (47.8%) |

## Supplementary Table S8. Missing current-state predictors at the earliest eligible index.

Missingness is measured before imputation in 386 development and 251 temporal patients. A latest value available by the index is used; this differs from index-date availability for the laboratory rules.

| **Predictor** | **Development missing n (%)** | **Temporal missing n (%)** |
| --- | --- | --- |
| Age | 0 (0.0%) | 0 (0.0%) |
| Sex | 0 (0.0%) | 0 (0.0%) |
| eGFR | 0 (0.0%) | 0 (0.0%) |
| Haemoglobin | 141 (36.5%) | 75 (29.9%) |
| Albumin | 164 (42.5%) | 94 (37.5%) |
| Potassium | 151 (39.1%) | 102 (40.6%) |
| Phosphate | 205 (53.1%) | 126 (50.2%) |
| Urea | 27 (7.0%) | 36 (14.3%) |

## Supplementary Table S9. Descriptive associations with predictor missingness.

One earliest horizon-evaluable index per patient is selected separately at each horizon. Positive counts refer to that selected index; they differ from patients positive at any repeated index. No hypothesis test identifies a missingness mechanism, and neither missing completely at random (MCAR) nor missing at random (MAR) is inferred from these summaries.

### Development — 12m

| **Predictor** | **State** | **n** | **Median age** | **Median eGFR** | **Positive indices** |
| --- | --- | --- | --- | --- | --- |
| Haemoglobin | missing | 91 | 55.0 | 61.0 | 3 |
| Haemoglobin | observed | 171 | 60.0 | 42.7 | 16 |
| Albumin | missing | 108 | 58.5 | 49.2 | 6 |
| Albumin | observed | 154 | 57.5 | 48.2 | 13 |
| Potassium | missing | 98 | 55.0 | 61.4 | 3 |
| Potassium | observed | 164 | 60.0 | 45.4 | 16 |
| Phosphate | missing | 139 | 56.0 | 60.1 | 4 |
| Phosphate | observed | 123 | 61.0 | 42.3 | 15 |
| Urea | missing | 18 | 67.0 | 45.9 | 1 |
| Urea | observed | 244 | 57.0 | 49.1 | 18 |

### Development — 24m

| **Predictor** | **State** | **n** | **Median age** | **Median eGFR** | **Positive indices** |
| --- | --- | --- | --- | --- | --- |
| Haemoglobin | missing | 84 | 55.0 | 62.0 | 7 |
| Haemoglobin | observed | 157 | 60.0 | 43.5 | 36 |
| Albumin | missing | 101 | 58.0 | 48.3 | 15 |
| Albumin | observed | 140 | 56.5 | 49.9 | 28 |
| Potassium | missing | 90 | 55.0 | 60.3 | 9 |
| Potassium | observed | 151 | 60.0 | 46.4 | 34 |
| Phosphate | missing | 129 | 56.0 | 60.1 | 15 |
| Phosphate | observed | 112 | 60.0 | 42.9 | 28 |
| Urea | missing | 16 | 67.0 | 45.9 | 1 |
| Urea | observed | 225 | 56.0 | 50.0 | 42 |

### Temporal — 12m

| **Predictor** | **State** | **n** | **Median age** | **Median eGFR** | **Positive indices** |
| --- | --- | --- | --- | --- | --- |
| Haemoglobin | missing | 45 | 54.0 | 69.8 | 2 |
| Haemoglobin | observed | 98 | 64.5 | 49.4 | 5 |
| Albumin | missing | 58 | 59.0 | 58.7 | 2 |
| Albumin | observed | 85 | 62.0 | 52.8 | 5 |
| Potassium | missing | 58 | 58.5 | 61.3 | 3 |
| Potassium | observed | 85 | 62.0 | 50.4 | 4 |
| Phosphate | missing | 68 | 57.0 | 62.9 | 3 |
| Phosphate | observed | 75 | 66.0 | 48.7 | 4 |
| Urea | missing | 20 | 69.0 | 55.7 | 0 |
| Urea | observed | 123 | 59.0 | 54.0 | 7 |

### Temporal — 24m

| **Predictor** | **State** | **n** | **Median age** | **Median eGFR** | **Positive indices** |
| --- | --- | --- | --- | --- | --- |
| Haemoglobin | missing | 26 | 58.0 | 65.5 | 3 |
| Haemoglobin | observed | 61 | 63.0 | 47.9 | 16 |
| Albumin | missing | 36 | 59.0 | 48.3 | 8 |
| Albumin | observed | 51 | 62.0 | 52.8 | 11 |
| Potassium | missing | 38 | 59.0 | 61.4 | 4 |
| Potassium | observed | 49 | 63.0 | 48.7 | 15 |
| Phosphate | missing | 43 | 59.0 | 61.9 | 4 |
| Phosphate | observed | 44 | 64.0 | 46.2 | 15 |
| Urea | missing | 11 | 69.0 | 55.7 | 0 |
| Urea | observed | 76 | 59.5 | 52.0 | 19 |

## Supplementary Table S10. Index eGFR source and paired agreement.

Counts cover all retained indices in the primary splits before horizon evaluability. Paired differences are reported eGFR minus calculated 2021 CKD-EPI eGFR, in mL/min/1.73 m². Repeated pairs are descriptive, not independent observations. Category boundaries are 15, 30, 45, 60 and 90. Historical laboratory equation identifiers were unavailable.

| **Measure** | **Development** | **Temporal** |
| --- | --- | --- |
| All indices | 1463 | 563 |
| Reported eGFR | 14 | 368 |
| Calculated eGFR | 1449 | 195 |
| Paired values | 14 | 368 |
| Patients contributing pairs | 14 | 183 |
| GFR-category disagreement | 1 | 43 |
| Median difference (Q1–Q3) | −0.73 (−1.24 to −0.04) | −0.64 (−2.25 to 1.93) |

## Supplementary Table S11. Temporal calibration of frozen risk components and outputs.

Calibration-in-the-large (CITL) fits only an intercept with logit(prediction) as an offset. The joint model fits both intercept and slope. Ideal CITL and joint intercept are 0, and ideal slope is 1. Probabilities were clipped to [10⁻⁶, 1−10⁻⁶] only for taking logits. Fits used unpenalized weighted likelihood. Confidence intervals are percentile intervals from 2,000 patient-cluster resamples; no recalibration was applied. All 2,000 fits per measure converged under the stated gradient and coefficient checks.

| **Measure** | **12m** | **24m coherent** | **24m direct** | **13–24m conditional** |
| --- | --- | --- | --- | --- |
| Patients | 143 | 87 | 87 | 80 |
| Event patients | 10 | 22 | 22 | 15 |
| Indices | 364 | 247 | 247 | 226 |
| Positive indices | 21 | 50 | 50 | 29 |
| Observed risk | 0.061 | 0.229 | 0.229 | 0.161 |
| Mean predicted risk | 0.067 | 0.199 | 0.193 | 0.122 |
| AUPRC | 0.510 | 0.677 | 0.672 | 0.483 |
| AUROC | 0.937 | 0.873 | 0.864 | 0.833 |
| Brier score | 0.042 | 0.116 | 0.121 | 0.109 |
| Quartile absolute error | 0.008 | 0.048 | 0.035 | 0.051 |
| CITL (95% CI) | −0.151 (−1.189, 0.596) | 0.273 (−0.316, 0.765) | 0.365 (−0.303, 0.947) | 0.395 (−0.241, 0.929) |
| Joint intercept (95% CI) | −0.158 (−1.376, 1.079) | 0.305 (−0.435, 1.272) | 0.129 (−0.584, 1.187) | 0.563 (−0.501, 1.811) |
| Slope (95% CI) | 0.996 (0.689, 1.640) | 1.028 (0.676, 1.661) | 0.820 (0.518, 1.397) | 1.107 (0.666, 1.865) |

The conditional evaluation excludes indices positive by day 365 and reweights within the remaining sample. It contains 80 patients, of whom 15 have at least one positive conditional index. Quartile error is the sum of bin weights multiplied by |observed−predicted risk|; it is not a smooth calibration error and is sensitive to binning. Near-ideal point estimates with wide intervals do not establish accurate individual probabilities.

Composition cannot lower p12. On the 24-month set, weighted mean p12 was 0.091 and mean coherent risk 0.199. Relative to the independently fitted direct 24-month model, 146/247 probabilities increased and 101/247 decreased; weighted mean direct risk was 0.193. Conditional q alone is not an uncombined 24-month cumulative probability because it is defined among first-year non-events.

## Supplementary Table S12. Event information in the original absolute-performance bootstrap draws.

Each of 2,000 draws samples patients with replacement and retains all indices. “Event-patient draws” counts duplicates; “distinct event patients” counts the original event patients represented at least once. These are event-information diagnostics, not an estimate of a formal effective sample size. Percentile values can be fractional.

| **Horizon** | **Quantity** | **Median** | **2.5–97.5 percentiles** | **Range** | **Zero draws** |
| --- | --- | --- | --- | --- | --- |
| 12m | event patient draws | 10 | 4.98–16.00 | 1–22 | 0 |
| 12m | unique event patients | 6 | 3.00–9.00 | 1–10 | 0 |
| 12m | positive index draws | 20 | 7.98–39.00 | 1–57 | 0 |
| 24m | event patient draws | 22 | 14.00–30.00 | 9–39 | 0 |
| 24m | unique event patients | 14 | 10.00–18.00 | 6–21 | 0 |
| 24m | positive index draws | 50 | 29.00–74.00 | 17–103 | 0 |

## Supplementary Table S13. Frozen-prediction performance restricted to complete temporal cases.

No model was refitted here. Complete cases have all eight current-state predictors at each evaluated index. This analysis changes the evaluation subset and therefore is not an unbiased comparison of imputation methods.

| **Horizon** | **Patients / event patients** | **Indices / positive** | **AUPRC** | **AUROC** | **Brier** |
| --- | --- | --- | --- | --- | --- |
| 12m | 64/5 | 144/11 | 0.540 | 0.962 | 0.043 |
| 24m | 40/12 | 100/22 | 0.578 | 0.806 | 0.153 |

## Supplementary Table S14. Simple documentation-ranking comparators.

All strategies rank the identical three temporally estimable labels on 50 positive-target indices from 30 patients. The other four candidate labels are excluded by the original estimability rule; none of these metrics evaluates a full seven-label deployment system. Recall@3 is necessarily 1. Development-prevalence ties follow the original label order; history ties use development prevalence. Prior mention uses all available earlier dates, whereas mention counts use the previous 365 days. Index-date text is excluded from these predictors.

| **Strategy** | **mAP@1** | **mAP@2** | **mAP@3** | **Recall@1** | **Recall@2** | **Recall@3** |
| --- | --- | --- | --- | --- | --- | --- |
| fitted model | 0.765 | 0.863 | 0.876 | 0.765 | 0.961 | 1.000 |
| development prevalence | 0.632 | 0.719 | 0.784 | 0.632 | 0.806 | 1.000 |
| prior mention | 0.756 | 0.858 | 0.871 | 0.756 | 0.961 | 1.000 |
| prior 365-day mention count | 0.843 | 0.902 | 0.915 | 0.843 | 0.961 | 1.000 |

| **Comparator** | **Metric** | **Model minus comparator** | **95% CI** |
| --- | --- | --- | --- |
| development prevalence | mAP@1 | 0.134 | 0.032 to 0.265 |
| development prevalence | mAP@2 | 0.145 | 0.068 to 0.228 |
| development prevalence | mAP@3 | 0.093 | 0.039 to 0.158 |
| prior mention | mAP@1 | 0.010 | −0.106 to 0.130 |
| prior mention | mAP@2 | 0.005 | −0.053 to 0.065 |
| prior mention | mAP@3 | 0.005 | −0.053 to 0.065 |
| prior 365-day mention count | mAP@1 | −0.078 | −0.229 to 0.075 |
| prior 365-day mention count | mAP@2 | −0.039 | −0.114 to 0.038 |
| prior 365-day mention count | mAP@3 | −0.039 | −0.114 to 0.038 |

Paired intervals use 2,000 patient-cluster draws with the primary patient weights. The count-based comparator has a higher mAP point estimate than the fitted model. No incremental value over prior-history ranking is established. This supports an exploratory documentation-review interpretation, not diagnostic inference.

## Supplementary Table S15. Fixed-specification missing-data sensitivity refits.

Algorithms, features, hyperparameters and development/temporal separation remain fixed. Primary reproduction matched stored probabilities within 10⁻⁷. Median-without-indicators removes indicator columns in both components. Complete-case refitting restricts each development task and temporal task to complete predictors. No alternative was selected using temporal performance; point estimates are exploratory and are not equivalence tests.

| **Variant / horizon** | **Temporal patients / events** | **Indices / positives** | **AUPRC** | **AUROC** | **Brier** |
| --- | --- | --- | --- | --- | --- |
| primary reproduction / 12m | 143/10 | 364/21 | 0.510 | 0.937 | 0.042 |
| primary reproduction / 24m | 87/22 | 247/50 | 0.677 | 0.873 | 0.116 |
| median without indicators / 12m | 143/10 | 364/21 | 0.496 | 0.939 | 0.042 |
| median without indicators / 24m | 87/22 | 247/50 | 0.679 | 0.870 | 0.116 |
| complete case refit / 12m | 64/5 | 144/11 | 0.496 | 0.954 | 0.048 |
| complete case refit / 24m | 40/12 | 100/22 | 0.577 | 0.819 | 0.154 |

Complete-case refitting used 117 development patients at 12 months and 89 development patients for the conditional component, versus 262 and 224 for the primary reproduction. Complete-case sample selection and model fitting both change, so differences cannot be attributed solely to imputation. Multiple imputation was not undertaken; the available data do not establish MAR, and neither tested strategy resolves potential missing-not-at-random (MNAR) bias.

## Supplementary Table S16. Exploratory clinical-history proxy comparator.

A fixed L2-logistic model (C=0.2, liblinear) with median imputation/indicators and standardization was fitted using development data only. Predictors were age, sex, current eGFR, any strictly prior diabetes/hypertension/kidney-disease mention, and current proteinuria positive/not-evaluable indicators. All predictors obey the report-time boundary. This is a locally derived comparator with documentation proxies, not an established risk equation. Medication and validated comorbidity ascertainment were unavailable.

| **Horizon** | **Patients / events** | **Indices / positives** | **AUPRC** | **AUROC** | **Brier** |
| --- | --- | --- | --- | --- | --- |
| 12m | 143/10 | 364/21 | 0.419 | 0.917 | 0.047 |
| 24m | 87/22 | 247/50 | 0.565 | 0.805 | 0.136 |

The comparison is descriptive and was added after review; it did not alter the locked models. The original paired comparison with the core model remains in main Table 3. Lower comparator point estimates do not establish superiority against comprehensive clinical prediction or KFRE.

## Supplementary Table S17. Availability of usable urine albumin-to-creatinine ratio (UACR).

The audit accepted explicitly identified urine microalbumin (mg/L) and urine creatinine (µmol/L), both valid and collected at exactly the same time, with results available by the index and collection within the preceding 365 days. The ratio was albumin/(urine creatinine/1000), in mg/mmol. There were 200 matched source pairs across the full source period. A test named albumin/creatinine but reported in mg/L was not accepted as a valid ratio: concentration units do not establish a ratio or justify assigning an assumed value.

| **Split / horizon** | **All indices** | **Usable indices** | **Usable patients** | **Event patients with UACR** | **Missing %** |
| --- | --- | --- | --- | --- | --- |
| Dev. / 12m | 1191 | 0 | 0 | 0 | 100.0 |
| Dev. / 24m | 1086 | 0 | 0 | 0 | 100.0 |
| Temporal / 12m | 364 | 9 | 5 | 2 | 97.5 |
| Temporal / 24m | 247 | 8 | 4 | 3 | 96.8 |

The 24-month subset contains only four temporal patients and three event patients, limiting comparisons with UACR-dependent KFRE versions. These counts describe usable historical measurements, not clinical albuminuria prevalence. Missing UACR was not set to normal. Because a published three-variable Tangri model does not require UACR, we evaluated its fixed age-sex-eGFR linear predictor separately in Supplementary Table S18.

## Supplementary Table S18. Exploratory comparison with the published three-variable Tangri model 2 linear predictor.

The fixed score used the published rounded hazard ratios from Tangri et al. [5]: log(0.75)×age/10 + log(1.46)×male sex + log(0.57)×eGFR/5. No coefficients were fitted locally and no temporal selection was performed. AUPRC and AUROC are patient weighted; differences are locked model minus published score, with 2,000 paired patient-cluster bootstrap samples. The comparison is restricted to ranking because a directly deployable horizon-specific baseline-risk equation for the three-variable model was unavailable and its treated-kidney-failure endpoint differs from the study composite. Brier score and calibration are therefore not reported.

| **Horizon / subset** | **Patients / events** | **Indices / positives** | **Metric** | **Locked** | **Tangri score** | **Difference (95% CI)** |
| --- | --- | --- | --- | --- | --- | --- |
| 12m / All temporal indices | 143 / 10 | 364 / 21 | AUPRC | 0.510 | 0.519 | −0.008 (−0.179 to 0.112) |
| 12m / All temporal indices | 143 / 10 | 364 / 21 | AUROC | 0.937 | 0.927 | 0.010 (−0.019 to 0.046) |
| 12m / eGFR 10–59 | 86 / 10 | 205 / 21 | AUPRC | 0.510 | 0.519 | −0.008 (−0.174 to 0.120) |
| 12m / eGFR 10–59 | 86 / 10 | 205 / 21 | AUROC | 0.890 | 0.873 | 0.017 (−0.033 to 0.075) |
| 24m / All temporal indices | 87 / 22 | 247 / 50 | AUPRC | 0.677 | 0.603 | 0.074 (−0.025 to 0.178) |
| 24m / All temporal indices | 87 / 22 | 247 / 50 | AUROC | 0.873 | 0.820 | 0.053 (0.003 to 0.119) |
| 24m / eGFR 10–59 | 55 / 20 | 149 / 46 | AUPRC | 0.698 | 0.630 | 0.069 (−0.039 to 0.186) |
| 24m / eGFR 10–59 | 55 / 20 | 149 / 46 | AUROC | 0.824 | 0.753 | 0.071 (−0.005 to 0.151) |

## Supplementary Table S19. Repeat-level model-selection stability audit.

The original patient-grouped folds and prespecified selection rule were reapplied separately in each of the five repeats. Values are median (range) across repeat-specific out-of-fold estimates. All repeats reuse the same development cohort; this audit is not nested selection-adjusted validation, a full model-selection bootstrap or external validation. It did not alter the locked models.

| **Component / output** | **Repeat-level selection** | **AUPRC** | **AUROC** | **Brier** |
| --- | --- | --- | --- | --- |
| 12m cumulative | Elastic net: 5/5 | 0.595 (0.578–0.631) | 0.887 (0.886–0.895) | 0.066 (0.064–0.067) |
| 13-24m conditional | Random forest: 5/5 | 0.385 (0.364–0.416) | 0.813 (0.802–0.823) | 0.105 (0.103–0.108) |
| Coherent 24m pair | Same pair: 5/5 | 0.713 (0.702–0.718) | 0.886 (0.881–0.890) | 0.107 (0.106–0.110) |
| Highest-AUPRC family / 12m | Current: 3/5; core: 2/5; longitudinal: 0/5 | — | — | — |
| Highest-AUPRC family / coherent 24m | Current: 5/5 | — | — | — |
| Longitudinal displacement gate / both horizons | Passed: 0/5 | — | — | — |

## Supplementary Table S20. Precision-based planning scenarios for future external validation.

Calculations used pmvalsampsize for a binary outcome following Riley et al. [23]. To satisfy the independent-observation assumption, inputs came from one earliest horizon-evaluable temporal index per patient: 7/143 events at 12 months and 19/87 at 24 months. The assumed observed-to-expected (O/E) ratio and calibration slope were 1; target total 95% CI widths were 0.20 for the O/E ratio and slope and 0.10 for the c-statistic. The linear predictor (LP; logit-risk) distribution was approximated as normal using the observed mean and standard deviation. Required size is the maximum of the three criteria. These are transparent scenarios for protocol planning, not retrospective power calculations or universal minima; a future study must update all inputs.

| **Horizon** | **Planning inputs** | **O/E criterion** | **Slope criterion** | **C-stat criterion** | **Final patients / events** |
| --- | --- | --- | --- | --- | --- |
| 12m | Prev 0.049; C 0.932; LP −4.296 (2.167) | 7470 | 4115 | 576 | 7470 / 366 |
| 24m | Prev 0.218; C 0.939; LP −2.111 (1.668) | 1376 | 2381 | 142 | 2381 / 520 |
